# Hybrid energy system optimization integrated with battery storage in radial distribution networks considering reliability and a robust framework

**DOI:** 10.1038/s41598-024-73808-8

**Published:** 2024-11-04

**Authors:** Mohammad Javad Aliabadi, Masoud Radmehr

**Affiliations:** Department of Electrical Engineering, Aliabad Katoul Branch, Islamic Azad University, Aliabad Katoul, Iran

**Keywords:** Batteries, Solar energy

## Abstract

This research presents a robust optimization of a hybrid photovoltaic-wind-battery (PV/WT/Batt) system in distribution networks to reduce active losses and voltage deviation while also enhancing network customer reliability considering production and network load uncertainties. The best installation position and capacity of the hybrid system (HS) are found via an improved crow search algorithm with an inertia weight technique. The robust optimization issue, taking into account the risk of uncertainty, is described using the gap information decision theory method. The proposed approach is used with 33- and 69-bus networks. The results reveal that the HS optimization in the network reduces active losses and voltage variations, while improving network customer reliability. The robust optimization results show that in the 33-bus network, the system remains resilient to prediction errors under the worst-case uncertainty scenario, with a 44.53% reduction in production and a 22.18% increase in network demand for a 30% uncertainty budget. Similarly, in the 69-bus network, the system withstands a 36.22% reduction in production and a 16.97% increase in load for a 25% uncertainty budget. When comparing stochastic and robust methods, it was found that the stochastic Monte Carlo method could not consistently provide a reliable solution for all objectives under uncertainty, whereas the robust approach successfully managed the maximum uncertainty related to renewable generation and network demand across different uncertainty budgets.

## Introduction

### Motivation and background

Hybrid energy systems with storage devices have increasingly been implemented to supply power to loads that are either vulnerable or located in remote areas, far from the grid. These systems provide a reliable energy solution in situations where extending traditional grid infrastructure is either challenging or economically unfeasible. Beyond off-grid applications, on-grid hybrid energy systems have also been deployed to enhance the flexibility and reliability of distribution networks, offering the potential for more efficient energy management and improved grid stability^1^. However, as the proportion of energy generated from renewable sources such as wind and photovoltaic (PV) systems continues to increase, managing their inherent variability and uncertainty has become a significant challenge. The intermittent nature of renewable energy, due to factors like weather conditions and time of day, introduces power fluctuations that can destabilize radial distribution networks, making it difficult to maintain the balance between supply and demand^2^. This uncertainty can lead to issues such as voltage deviation and increased power losses, both of which can undermine the overall performance of the network. Distribution network operators (DNOs) must account for these uncertainties in their planning and operation strategies. Proper forecasting tools and uncertainty models are essential for predicting power generation and understanding how fluctuations in renewable output will impact the network^3,4^.

### Literature review

Numerous investigations have been conducted regarding the most efficient distribution network resource allocation for energy. The authors of^5^ describe how the ant lion algorithm (ALO) is utilized to optimize the hybrid size and placement of wind turbines (WTs) and PVs in the network. A method for designing the most effective system with the use of distributed generation (DG) systems is offered in^6^. This method utilizes the wild horse algorithm (IWHO) to achieve the following objectives: peak energy conservation, reducing loss, and enhancing the voltage profile. The authors of^7^ discuss the most efficient way to distribute a renewable energy source (RES) that can provide both active and reactive power to the network while minimizing system losses. This research investigation introduces an effective optimization method known as the Honey Badger Algorithm (HBA) in reference^8^. The HBA is utilized to ascertain the ideal dimensions and placement of capacitors and various types of DG in order to minimize the active loss across the entire network. The authors of^9^ use an improved genetic algorithm to find the best size and position of the battery system and PV production system to minimize losses, enhance the bus voltage profile, and decrease the voltage imbalance of the network. A hybrid optimization approach is introduced in reference^10^ that combines particle swarm optimization and the genetic algorithm technique to determine the most efficient way to allocate WTs in the network. An investigation was conducted in^11^ into the design of the active network, taking into account the uncertainty associated with distributed wind energy production. Additionally, the hybrid particle swarm algorithm was implemented to resolve the issue. The method to identify the optimum number of WTs to minimize network losses is described in^12^. The GA is employed to optimize the system, while load dispersion is utilized to compute energy losses. Using the artificial bee colony (ABC) algorithm,^13^ describes the deployment and planning of hybrid diesel-PV DG resources in the network in an attempt to minimize power and cost losses.

In^1^, the optimal design of a hybrid photovoltaic-wind generator system with battery storage with off-grid and on-grid operation modes is presented to supply annual load demand considering environmental emissions and energy generation cost, as well as the cost of load losses using a spotted hyena optimisation (SHO). In^14^, the study explores the allocation of hydrogen stations and electric vehicle charging within distribution networks to optimize microgrid performance. A two-stage stochastic model is employed, utilizing a multi-objective approach to address both economic and environmental considerations in energy management. In^15^, an optimal designing and energy management of hybrid photovoltaic/wind/fuel cell (PV/WT/FC) system is presented with cost of hybrid system life span minimizing and considering loss of load interruption probability using a sine–cosine algorithm (ISCA). In^16^, the study presents the design and optimization of a biomass-powered cogeneration plant integrated with a heat recovery unit, taking into account a compressed air energy storage system. A multi-objective genetic algorithm is employed to achieve optimal performance. In^17^, optima planning of an island sustainable system that consists of a wind turbine, battery, combined heat and power system, and thermal storage is developed to meet electrical and thermal loads at the same time to minimize the planning cost using a hybrid optimization algorithm. In^18^, a hybrid system consisting of wind, photovoltaic, diesel, and battery energy storage is designed using a combination of the sine–cosine and crow search algorithms to minimize the total planning cost of energy resources and storage, while also reducing emission costs for an optimal robust structure. In^19^, the optimal design of a hybrid photovoltaic-battery-diesel system in China is introduced, utilizing an improved Henry gas solubility optimizer to determine the ideal component sizing for minimizing system costs. In^20^, an economic scheduling approach for microgrids, incorporating renewable energy sources and based on the energy hub model, is presented. This approach uses demand response and an improved water wave optimization algorithm for participation in energy market operations, with a focus on demand response (DR).

The objective of the study conducted in reference^21^ was to optimize the network’s wind and PV energy resource allocation and planning to minimize production costs, enhance reliability, and reduce losses associated with the fluctuating usage arrangement caused by the COVID-19 pandemic. The primary aim is to determine the optimal operational point, which comprises the optimal placement and dimensions of energy resources, even when faced with the most severe load shifting circumstances. This is accomplished by employing the Turbulent Water Flow Algorithm (TFWO), which guarantees the highest network efficiency amidst the COVID-19 pandemic. A probabilistic methodology was implemented to account for production uncertainty in the setting of the COVID-19 pandemic. An effective method was presented in^22^ for identifying the best size and location of renewable power generation generators in networks, taking into account uncertainties in demand and power production. The Artificial Hummingbird (AHA) algorithm was utilized in this endeavor to minimize the anticipated total cost. Additionally, the publication fee is assessed. An investigation was conducted in^23^ into the placement of clean sources utilizing evolutionary programming (EP) while load uncertainties and DG sources were taken into account. The aim is minimizing the losses and enhance the voltage condition. A quasi-sequential Monte Carlo approach is introduced in reference^24^ to assess the effects of extensive PV power usage on customer node reliability as well as system energy and reserve utilization. The violent method was first proposed in reference^25^ as a solution to the probabilistic optimal load distribution problem. In comparison to the Monte Carlo simulation results, the Camiolent method yielded results that decrease in the computational burden while maintaining a high degree of accuracy. A hybrid probabilistic-feasibility assessment tool was developed in^26^ by combining the fuzzy method and Monte Carlo simulation to examine the impact of non-deterministic power generation from renewable distributed generators (DGs) on the active losses of networks. The authors of^27^ utilized the Monte Carlo method to assess the system’s reliability in the face of wind farm power uncertainty for a variety of distributions, including the Weibull distribution. The matter of power plant emissions (UC) was examined in^28^ in an effort to reduce operating expenses in light of the unpredictability of wind energy production. Utilizing the the information gap decision theory (IGDT) method, the uncertainty of wind energy has been assessed. The impact of variations in load on operation cost has also been investigated. The results collected demonstrate that the suggested approach is effective in assessing the UC issue when the wind power uncertainty is considered.^29^ describes an approach for preventive and restorative voltage control in automated power systems. An uncertainty-aware approach with the IGDT is implemented, which employs opportunity-seeking and risk-averse techniques. The outcomes of this approach have enabled the selection of the resistance level in consideration of the optimal uncertainty budget. A reliability-based singular collaboration is resolved in^30^, and the resulting solutions are subsequently utilized to the settlement of the reservation market with non-deterministic dynamic loads via the IGDT. By employing this approach, the person in charge of the system can determine the optimal course of action by considering the supply side’s uncertainty and the intended level of risk. The IGDT for resilient planning of smart apartment buildings in the context of price uncertainty is explained in^31^. Consequently, proprietors of smart homes in condominiums, which are categorized as small-scale loads, can employ the aforementioned theory to enhance their decision-making process in the face of price volatility. A novel framework is presented in^32^ that aims to optimize the effectiveness of an energy pole comprising fuel cell vehicles, renewable energy sources, and PHEVs. The framework employs the IGDT technique to model the uncertainty associated with the power used by PHEVs while on journeys, taking into account risk aversion and risk-seeking strategies. incorporate the uncertainty integrated with the power utilization of electric vehicles through the use of IGDT enables the operator of the energy pole to optimize the pole’s functioning in light of potential shifts in PHEV electrical usage.

## Research gaps

Despite the growing capacity for microgrid production, most research on optimal microgrid architecture heavily relies on meteorological data to account for variations in renewable energy generation. Many studies have employed the Monte Carlo Simulation (MCS) method to model uncertainty. MCS is widely regarded as a reliable and precise stochastic technique, requiring data to follow a specific probability distribution that reflects the characteristics of the modified data. This distribution must have the same mean and variance as the original dataset. During the prediction process, values are randomly selected from the probability distribution to account for uncertainty^33^. However, while MCS is effective in handling uncertainties with known statistical properties, it may not be suitable for scenarios where parameter uncertainties are ambiguous or hard to quantify. On the other hand, this method is based based on probability distribution function (PDF) of defined scenarios and is very time-consuming.

Recently, the Information Gap Decision Theory (IGDT)^34^ has gained attention as an alternative method for modeling uncertainty in the systems . Unlike MCS, IGDT is a non-probabilistic, and fast-solving, robust approach that focuses on making decisions under uncertainty by identifying the maximum uncertainty radius in worst-case scenarios. This allows system planners to design solutions that are resilient to errors in forecasting, even when the precise nature of the uncertainty is unknown. IGDT’s ability to enhance system resilience under severe uncertainty makes it particularly useful in the context of hybrid energy systems, where renewable generation and network load uncertainties can lead to significant performance challenges. By incorporating IGDT into the optimization of hybrid energy systems integrated with battery storage, researchers can develop more robust frameworks that safeguard against the worst impacts of forecast errors and variability in renewable energy production. This highlights a key research gap in the area of hybrid energy system optimization: the need for more robust uncertainty modeling techniques that go beyond probabilistic approaches like MCS, especially in the face of increasing renewable energy penetration in radial distribution networks.

### Contributions

The IGDT is a highly effective instrument for assessing and contrasting schemes implemented during periods of uncertainty. By utilizing this approach, the decision maker can analyze the effectiveness of each strategy, ascertain their priorities, and assess its anticipated objective function. Conversely, robust optimization techniques, such as the IGDT, necessitate the inclusion of the system’s worst-case scenario to determine its optimality; in this regard, the uncertainty parameters consistently remain within the uncertainty set. In contrast to the random Monte Carlo approach, the robust approach guarantees the system’s robustness^34^.

The following are the contributions made by this article in response to the identified research gaps:

- A robust optimization structure for allocating the hybrid PV/WT/Battery energy systems in radial 33-, and 69-bus distribution network considering production and load uncertainties
- Determination of the maximum uncertainty radius uncertain parameters using the robust structure, and improved crow search algorithm
- Implementing a multi-criteria objective function that incorporates subscriber reliability enhancement and the minimization of power losses and voltage deviations
- Assessing the system’s robustness in the most severe uncertainty scenario
- Assessing the degree of risk assumed by the system through the evaluation of uncertainty parameters

### Paper organization

In following the HS modeling, and operation is presented. Then the proposed methodology of the problem with objective function, and restriction are developed. After this, the proposed optimizer is formulated. Then the robust framework based on the IGDT is presented. The simulation results are given, and finally the findings are analyzed and concluded.

### Modeling of the studied HS

**Fig. 1:**
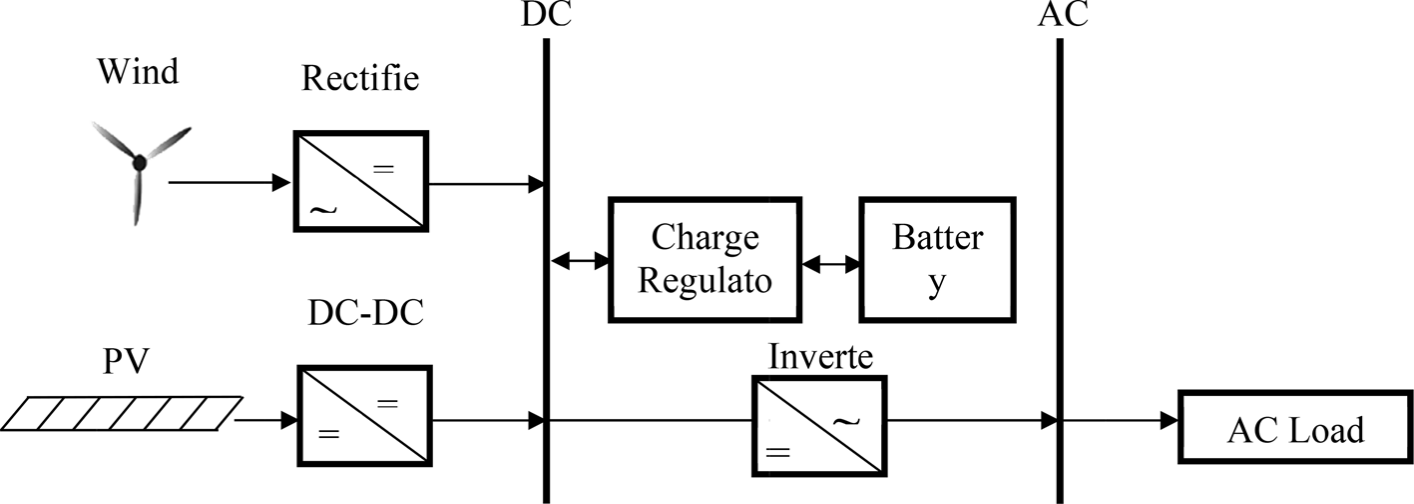
Schematic of the hybrid PV/WT/Batt system.

This study presents a hybrid PV/WT/Batt system, as depicted in Fig. 1. The system is constructed around a battery storage system and consists of WTs and PVs serving as the primary power supplies. A battery bank is utilized for the storage system, along with a converter and regulator. When the system is operating at a surplus of power, the battery serves as an energy storage medium to offset any deficiencies in system power.

The operation strategy of a hybrid PV/WT/Batt system can be structured around two key scenarios: surplus power and deficit power. These strategies ensure that the system operates efficiently and can manage the variability of renewable generation and the energy demands of the load.

- Extra Power (Battery in Charge State)

In the event that the combined power generated by the photovoltaic panels and wind turbines exceeds the energy demand of the load, the extra power injected to the batteries for charging and also to the distribution network for a specified bus.

- Deficit Power (Battery in Discharge State)

When the power generated by the photovoltaic and wind energy systems is insufficient to meet the load demand, the system enters the deficit power mode, where the battery discharges to supply the shortfall. In this condition no power injected to distribution network.

- Power Balance (Battery in Neutral State)

When the power generated by the photovoltaic and wind energy systems is equal to the load demand, the system is not in extra and shortage power conditions and all renewable power generated will inject to the load demand and also no power injected to the batteries, and distribution network.

### PV power model

It is possible to calculate the power generated by individual PVs about the quantity of radiation by employing the subsequent equation^35–37^.

1 $$P_{PV} (t) = P_{PV - Rated} \times \frac{G}{{G_{ref} }} \times (1 + K_{T} (T_{c} - T_{ref} ))$$

where, G denotes the radiation perpendicular to the array’s surface in units of W/m2. P_PV-Rated_ signifies the nominal power output of each PV panel, G_ref_ corresponds to 1000 W/m2. Moreover, K_T_ represents the coefficient of temperature, -0.0037 per ^0^C for the PV panel. T_ref_ is the PV cell standard condition temperature.

### WT power model

When the wind speed surpasses the cut-in speed (Vci), the WT initiates the generation process. When the wind speed exceeds the nominal speed of the WT, the generated electricity remains consistent. However, if the wind speed surpasses the cut-out speed (Vco), the WT will cease operation as a precautionary measure. The power of individual wind generators is calculated by the wind speed and the subsequent equation^35–37^.

2 $$P_{WT}^{{}} = \left\{ {\begin{array}{*{20}l} {0;} \hfill & {v \le v_{ci} ,\,v_{{}} \ge v_{co} } \hfill \\ {P_{WT - Rated} \times \left( {\frac{{v_{{}} - v_{ci} }}{{v_{r} - v_{ci} }}} \right);} \hfill & {v_{ci} \le v_{{}} \le v_{r} } \hfill \\ {P_{WT - Rated} ;} \hfill & {v_{r} \le v_{{}} \le v_{co} } \hfill \\ \end{array} } \right.$$

where, v is the wind speed. P_WT_ is the specified WT power, while Vci, Vco, and Vr represent the low cutoff speed, high cutoff speed, and nominal speed of the WT, respectively.

### Battery model

The dynamic characteristics of PVs and wind generators cause the battery bank system capacity to fluctuate continually. The battery state of charge (SOC)^37^ in such a system is as follows.

- In instances where the power of PVs and WTs surpasses the demand, the battery bank enters charging mode. It is assumed that 50% of the discrepancy between the power produced and the demand is transferred to the battery bank for charging purposes, while the remaining portion is distributed through the network. The quantity of charge present in the battery bank at time t can be calculated using the subsequent equation:

3 $$\begin{aligned} P_{Batt} (t) & = P_{Batt} (t - 1) + 0.5 \\ & \quad *\left[ {(N_{PV}^{{}} \times P_{PV}^{{}} (t) + N_{WT}^{{}} \times P_{WT}^{{}} (t)) - \frac{{P_{Load}^{{}} (t)}}{{\eta_{INV} }}} \right] \\ \end{aligned}$$

In the given context, P_Batt(t)_ and P_Batt(t-1)_ are the charge values of battery at t and t-1, respectively. The burden is denoted as P_load(t)_, while $\eta_{INV}$ represents the inverter’s efficiency and N_PV_ and N_WT_ are PVs and WTs number, respectively.

- When the combined power produced by PVs and WTs falls short of the demand, the battery enters a discharge mode. The battery efficiency is thought to be one hundred percent in this investigation. Hence, at time t, the charge quantity of the battery bank can be defined by

4 $$\begin{aligned} P_{Batt} (t) & = P_{Batt} (t - 1) \\ & \quad - \left[ {\frac{{P_{Load}^{{}} (t)}}{{\eta_{INV} }} - (N_{PV}^{{}} \times P_{PV}^{{}} (t) + N_{WT}^{{}} \times P_{WT}^{{}} (t))} \right] \\ \end{aligned}$$

### HS cost model

The present value cost (NPC) of the HS is computed as follows^35–37^: it comprises investment cost (C_INVESTC_) and maintenance cost (C_MAINC_) over the system’s effective life:

5 $$NPC = C_{INVESTC} + C_{MAINC}$$

The investment cost is computed in the following manner: WTs, PVs, battery banks, and inverters are all components of the investment expense.

6 $$C_{INVESTC} = (C_{PV} \times N_{PV} ) + (C_{WT} \times N_{WT} ) + (C_{Batt} \times N_{Batt} ) + (C_{INV} \times N_{INV} )$$

where, C_PV_ represents the PV unit cost, C_WT_ denotes the WT unit cost, C_Batt_ signifies the battery bank unit cost, and CINV signifies the inverter unit cost. N_PV_ represents the PVs number, N_WT_ the number of WTs, N_Batt_ is the batteries number, and N_INV_ the inverters number.

Using the subsequent formula, one can determine the cost of system maintenance for the equipment.

7 $$C_{MAINC} = C_{PV,M} \times N_{PV} + C_{WT,M} \times N_{WT} + C_{Batt,M} \times N_{Batt}$$

In this context, C_PV,M_, C_WT,M_, and C_Batt_,M represent the yearly expenses associated with maintaining PV panels, WTs, and batteries, respectively. Maintenance fees for inverters are eliminated.

## Methodology

The forthcoming section provides the formulation of the optimization issue about HS in the network. The restrictions and target function of the problem are delineated in this particular section.

### Objective function

The goal of this issue is to integrate the HS into a network for minimizing the losses, voltage oscillations, and network energy not-supplied. Additionally, the network’s reliability is improved by reducing the unsupplied energy of customers (ENS) caused by network line outages. This is accomplished in a multi-objective manner using the weighted coefficients approach.

8 $$F_{{}}^{OF} = w_{1} \times \left( {F_{1}^{OF} /F_{1,\max }^{OF} } \right) + w_{2} \times \left( {F_{2}^{OF} /F_{2,\max }^{OF} } \right) + w_{3} \times \left( {F_{3}^{OF} /F_{3,\max }^{OF} } \right)$$

where, $F_{1}^{OF}$, $F_{2}^{OF}$ and $F_{3}^{OF}$ denote the loss function, the voltage deviation function, and the reliability enhancement function, respectively. $F_{1,\max }^{OF}$, $F_{2,\max }^{OF}$ and $F_{3,\max }^{OF}$ represent the maximum value of voltage deviations, losses, and ENS, respectively. and $w_{1}$, $w_{2}$ and $w_{3}$ are each component weight of the goal function. for which the weight of each component is such that the values summation of absolute of the aforementioned weights should be equal one. The goal functions representing losses and voltage oscillations are provided in the subsequent section.

### Power losses

Active power loss minimizing represents one of the considerations when integrating an HS into the network. The definition of the loss relationship is defined by ^5,6^

9 $$F_{1}^{OF} = P_{Loss} = \sum\limits_{i = 1}^{{N_{branch} }} {R_{i} } \times \left| {I_{i} } \right|^{2}$$

The variables denoted as |Ii|, the current in the i-th branch, P_Loss_ is total losses, R_i_ is resistance of the ith line, and N_branch_ denptes the number of lines.

### Voltage profile

The enhancement of the network voltage profile is an outcome of the mitigation of variations in the bus voltage. The following is the definition of the target function for decreasing the voltage variations of network buses^38^:

10 $$F_{2}^{OF} = VD = \sqrt {\frac{1}{{N_{bus} }} \times \sum\limits_{i = 1}^{{N_{bus} }} {(v_{i} - v_{p} )^{2} } }$$

11 $$v_{p} = \frac{1}{{N_{bus} }} \times \sum\limits_{i = 1}^{{N_{bus} }} {v_{i} }$$

where, VD denotes the voltage profile index, v_i_ signifies the voltage of the i-th bus, v_p_ represents the buses’ mean voltage, and N_bus_ signifies the number of buses.

### Reliability

Strengthening reliability is a fundamental objective in the operation of networks^39^. Improving network reliability is characterized in this study as reducing the amount of unsupplied energy (ENS) exhibited by network subscribers. A presentation of load points utilizing basic measures of energy consumption in ENS is computed by

12 $$ENS = \sum\limits_{i = 1}^{{N_{L} }} {\sum\limits_{j = 1}^{{N_{l} }} {RP_{i} \times LL_{i} \times TR_{i} } }$$

where, ENS represents the network unsupplied demand, N_L_ signifies the count of loads disconnected as a result of the line i’s outage, LL_i_ indicates the length of line i, TR_i_ represents the repair period for line i, and RP_i_ refers to the probability of line i’s outage amount.

### Constraints

The optimization procedure takes into account the following constraints^38,39^^and^^5,6^:

- The lowest and greatest equipment counts for HSs

13 $$N_{i - \min } \prec N_{i} \prec N_{i - \max }$$

where, N_i_ is the number of equipment i, N_i-min_ and N_i-max_, the lower and upper number of equipment i includes the PVs, WTs, and batteries number.

Lowest and greatest amount of battery bank charge

Where, N_i_ denotes the quantity of equipment i, while N_i-min_ and N_i-max_ represent the minimal and maximum quantities of equipment i, respectively, which comprise the PVs, WTs, and batteries number.

- The lowest and greatest charge levels of the battery bank

14 $$P_{Batt - \min } \le P_{Batt} \le P_{Batt - \max }$$

where, P_Batt-max_ denotes the utmost charge value that can be applied to the battery and is equivalent to the rated capacity (S_Batt_) of the battery. The battery bank’s minimum charge value attained during the maximum discharge depth (DD) is denoted as P_Batt-min_^37^.

15 $$P_{Batt - \min } = (1 - DD) \times S_{Batt}$$

- Bus voltage

16 $$V_{\min } \le V_{i} \le V_{\max } ;\quad {\text{i}} = 1,2, \ldots {\text{N}}_{{{\text{bus}}}}$$

- Permitted the flow of current by means of every network branch

17 $$I_{\min } \le I_{i} \le I_{\max } ;\quad {\text{i}} = 1,2, \ldots {\text{N}}_{{{\text{branch}}}}$$

- Maximum output of every renewable DG

18 $$0 \le P_{DGi} \le P_{{DG\,\max_{i} }} ;\quad {\text{i}} = 1,2, \ldots {\text{N}}_{{{\text{DG}}}}$$

- Power balance

19 $$P_{Post} + \sum\limits_{i = 1}^{{N_{HS} }} {P_{HS} (i) = } \sum\limits_{i = 1}^{l} {P_{Lineloss} (i) + \sum\limits_{q = 1}^{N} {Pd(q)} }$$

where, V_min_ and V_max_ represent the lowest and highest voltages of bus, N_bus_ signifies the buses number, I_min_ and I_max_ denote the lowest and highest line current sizes, P_DGi_ and P_DGmaxi_ signifies the DG power and greatest DG size, respectively, and P_Lineloss_ denotes the power missed in the grid branches, P_HS_ is the power inserted from the HS to the grid, P_d_ indicates grid load, and P_Post_ is delivered power of the post to the grid. Additionally, N_HS_ denotes the quantity of HSs that comprise the network.

In the optimization process, if any of the constraints (Eqs. (13)-(19)) of the problem are violated, a penalty function will be added to the objective function for each of them. In this way, the optimization objective function is bound and optimized under the constraints of the problem.

### Optimization method

The optimization problem in this study is non-linear due to the integration of renewable energy sources and battery storage systems. Also, the stochastic nature of renewable generation and network load uncertainties introduces additional complexity. Moreover, the use of IGDT adds another layer of non-linearity, as IGDT seeks to detremine system robustness under uncertain conditions, often leading to non-convex optimization landscapes. Metaheuristic algorithms are preferred for solving such non-linear and non-convex problems because they do not rely on gradient information or assumptions of convexity. In contrast, mathematical programming methods like linear programming are effective only for linear or convex problems. Metaheuristics provide the advantage of exploring a wider search space and finding good approximations of the global optimum, even when the problem is non-convex or involves uncertainties and constraints that are difficult to handle analytically.

**Fig. 2:**
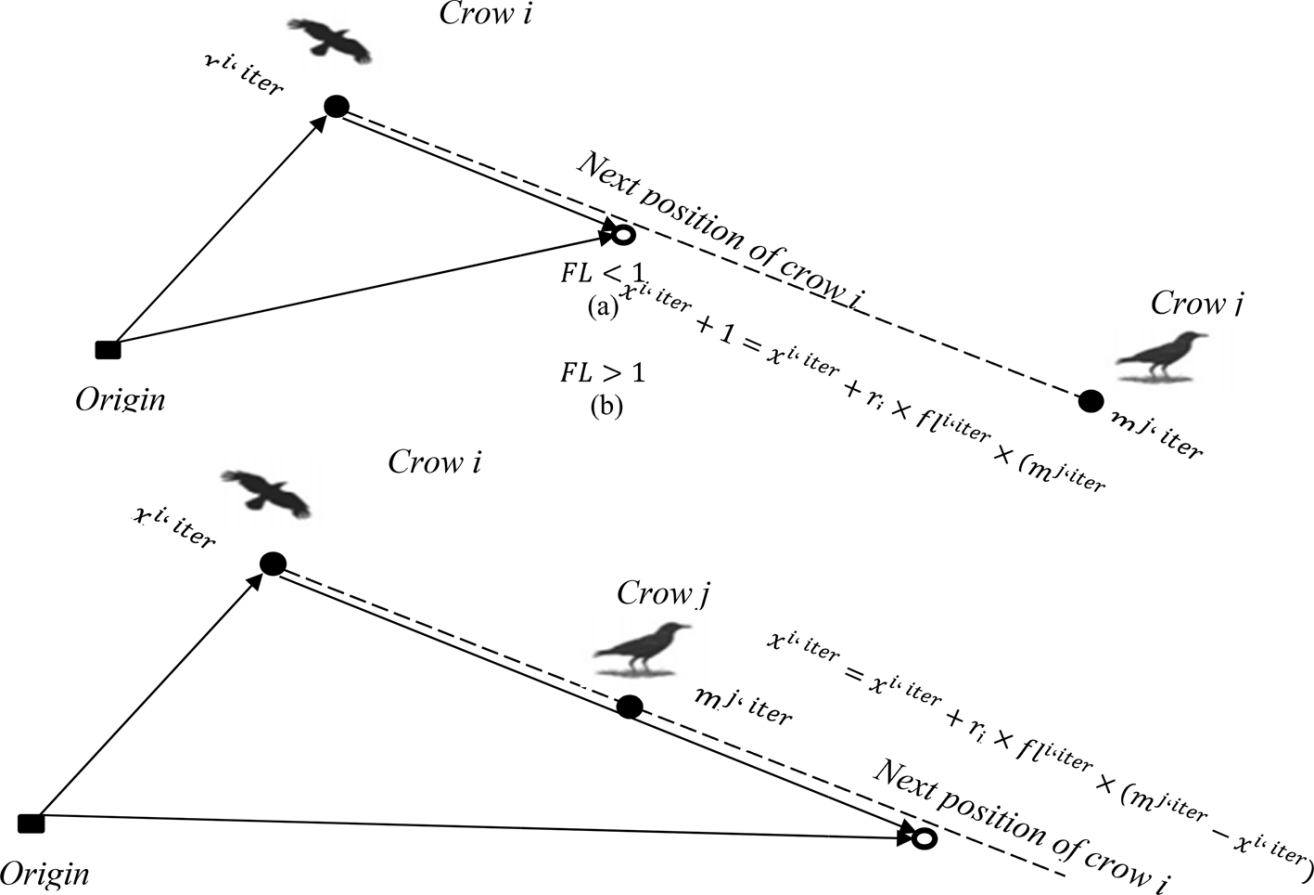
Crow search process across two functional logic (FL) phases.

In this research, the Improved Crow Search Algorithm (ICSA), a meta-heuristic technique, was employed to tackle the problem at hand. This method draws inspiration from the notably intelligent behavior of crows, as outlined in the original paper referenced as^40^. Known for having the highest brain-to-body ratio among birds, crows exhibit intelligence levels just below that of humans. They have the ability to recognize faces, alert others to the presence of unfamiliar individuals, and utilize tools to retrieve food hidden in various locations, remembering these spots for months. Crows, being opportunistic, often observe their peers to discover superior food sources, a trait driven by their natural inclination. The process of locating concealed food involves two distinct phases influenced by whether the crow being followed is aware of the pursuit. In the first phase, the oblivious crow leads its follower to the stash, initiating a search in a localized area. In phase two, when the crow becomes cognizant of being observed, it triggers a shift to a more randomized search pattern across a broader area, as detailed in^40^. This behavior is graphically represented in Fig. 2, highlighting the dual-phase logic underpinning the crow search algorithm’s functionality.

For a total of N crows, the ith crow position during the iter iteration is defined as follows, according to^40^:

20 $${\text{X}}^{{\text{i,iter}}} = ({\text{i}} = 1,2, \ldots ,{\text{N}};\,\,{\text{iter}} = 1,2, \ldots ,{\text{iter}}_{\max } )$$

where, ${\text{X}}^{\text{i},\text{iter}}=\left[{\text{x}}_{1}^{\text{i},\text{iter}},{\text{x}}_{2}^{\text{i},\text{iter}},\dots .,{\text{x}}_{\text{d}}^{\text{i},\text{iter}}\right],{\text{iter}}_{\text{max}}$ is the greatest number of iterations, and it’s presupposed that the subsequent dimension, d, corresponds to the number of crows. Each crow retains its optimal experience in memory. During subsequent iterations, this prior position is recalled and communicated by ${m}^{j,iter}$. The crows navigate within their vicinity, exploring these areas to discover food sources surpassing their current reserves.

The CSA initiates with an array of random solutions, thereafter embarking on a quest to identify the best solution within the problem domain through memory updates and leveraging the awareness probability (AP), thereby generating a new position. Essential CSA parameters consist of the flight length (FL) and AP. Based on AP, the CSA delineates two distinct phases as follows:

Phase 1: Crow j does not realize that it is being pursued by crow i. Consequently, crow i moves closer to the location where crow j has concealed its food source (as shown in Fig. 2 and a). During this phase, the updated location of crow i can be described by^40^

21 $$x^{i,iter + 1} = { }x^{i,iter} + r_{i} { } \times FL^{i,iter} \times \left( {m^{j,iter} - x^{i,iter} } \right)$$

where, ${r}_{i}$ represents a uniformly distributed random ranging from 0 to 1, and ${FL}^{i,iter}$ signifies the distance flown by crow i during the iteration iter.

Phase 2: Crow j realizes it is being pursued by crow i. To safeguard food source is pilfered by crow i, it misleads crow i by altering its location within the search area.

Thus, stages 1 and 2 are reformulated in the following manner^40^:

22 $$x^{i,iter + 1} = \left\{ {\begin{array}{*{20}l} {x^{i,iter} + r_{i} \times FL^{i,iter} \times \left( {m^{j,iter} - x^{i,iter} } \right)} \hfill & {r_{j} \ge AP^{j,iter} } \hfill \\ {a\,random\,position} \hfill & {otherwise} \hfill \\ \end{array} } \right.$$

Here, ${x}^{i,iter}$ denotes the present location of crow i during the iteration iter, ${r}_{j}$ is a uniformly distributed random number from 0 to 1, and ${AP}^{j,iter}$ indicates the awareness probability of crow j within the same iteration iter.

With the increasing complexity and dimensions of the optimization problem, the CSA can experience early convergence and become trapped in local optima. To address this issue, this study has enhanced the performance of the CSA under these conditions. The method of reduced inertia weight (IW)^15^ is applied to improve the conventional CSA for better performance in both global and local searches, resulting in the improved version known as ICSA. The effectiveness of the CSA is augmented by adjusting the value of φ. The IW value decreases in a nonlinear manner from ${\upphi }_{max}$ to ${\upphi }_{min}$. A larger value of ϕ facilitates global heuristic search capabilities, while a smaller value of ϕ supports efficient local discovery searches. ϕ is defined in the following way:

23 $$\phi = \phi_{min} + \left( {0.5 + 0.5 \times \cos \left( {\frac{\pi \times iter}{{iter_{max} }}} \right)} \right)^{\lambda } \times \left( {\phi_{max} - \phi_{min} } \right)$$

where, ${\upphi }_{max}$ and ${\upphi }_{min}$ are the greatest and lowest quantities of ϕ, respectively. ${iter}_{max}$ denotes the total number of iterations, and λ is a constant value set at λ = 10. Equation (24) is reformulated using the decreasing inertia weight method in the following manner:

24 $$x^{i,iter + 1} = { }\left\{ {\begin{array}{*{20}l} {\phi \times x^{i,iter} + r_{i} \times FL^{i,iter} \times \left( {m^{j,iter} - x^{i,iter} } \right)} \hfill & {r_{j} \ge AP^{j,iter} } \hfill \\ {a\,random\,position} \hfill & {otherwise} \hfill \\ \end{array} } \right.$$

### Deterministic optimization implementation

The steps for the HS optimization in networks are carried out as follows:

- Step 1: Input Data Introduction. At this phase, input data such as bus load information and network lines, costs related to investment, maintenance, and replacement of system equipment, alongside data on load, solar radiation, wind speed, rated power, and efficiency of system equipment, population size, and iteration count are specified. In this research, the algorithm’s population is set at 50, with a maximum iteration limit of 200. The selection of population size and iteration limit is based on achieving optimal accuracy, convergence speed, and the problem’s best objective function, determined through trial and error.
- Step 2: Initial Population Generation. Here, the firstly population is created within the search space randomly, establishing the decision variables’ vector which includes the optimum placement of the HS, the PVs, WTs, and batteries number.
- Step 3: Energy Management System. This step involves implementing the strategy for system usage.
- Step 4: Objective Function Evaluation and Constraint Verification. The problem’s objective function is evaluated against the system equipment and network operational constraints. The optimal set of variables correspond to the lowest target function is identified.
- Step 5: Population Update and Random Variable Selection using the ICSA.
- Step 6: Objective Function Re-evaluation and Constraint Re-verification. If the target function value obtained in step 6 surpasses that from step 4, it is updated accordingly, and the associated variable set is selected the optimal set.
- Step 7: Convergence Condition Check. This step determines whether the best objective function value and the maximum iteration have been reached. If so, proceed to step 8; otherwise, return to step 5.
- Step 8: The ICSA Termination and Result Documentation.

### Robust optimization implementation

IGDT, which stands for Info-gap Decision Theory, is a non- non-fuzzy and non-probabilistic approach for managing quantities under uncertainty. It proves particularly useful in scenarios characterized by high uncertainty or when data is insufficient. This method assists decision-makers in expanding or contracting the range of uncertainty they are willing to accept in relation to a forecasted target value. This ensures that the forecasted value does not fall below a predefined minimum outcome.

A Risk-averse (RA) decision-making strategy is employed to extend the scope of uncertain parameters in a manner that accounts for potential deviations from the initial data, guaranteeing that the outcomes of the model are not inferior to the anticipated value. The objective function’s value in a robust problem setup meets the criteria based on the uncertainty budget within the optimal range of uncertainty parameter fluctuations as follows:

25 $$\begin{aligned} & \hat{\alpha }\left( {d,B_{c} } \right) = \max \left\{ {\alpha :\left( {\mathop {\max }\limits_{{X \in U(\alpha ,\overline{X})}} OF_{Deterministic} \left( {X,d} \right) \le OF_{Robust} } \right)} \right\} \\ & subjected\,\,to\,\,OF_{Robust} = (1 + \sigma )OF_{Deterministic} ,\,\,\sigma \in [0,1) \\ \end{aligned}$$

The robustness function, utilizing the IGDT approach, is defined within certain constraints. Here, $\hat{\alpha }(.)$ indicates the maximum allowable deviation for the variable under uncertainty. $OF_{Deter\min istic}$ refers to the basic objective function (the outcome derived from the deterministic problem devoid of uncertainty), and $OF_{Robust}$ represents the target function value in the robust problem as determined by the uncertainty budget $\sigma$. $\sigma$ signifies the level of risk deviation or the uncertainty budget as chosen by the decision-maker within the RA (Risk-averse) strategy.

Under the RA strategy, the framework is built upon the IGDT-based robust function, with $\alpha_{{{\text{Re}} n}}^{c}$ and $\alpha_{Ld}^{c}$ essentially capturing the utmost boundary of uncertainty or the degree of robustness against any uncertainties. In this scenario, the solution decided upon through the RA model, leveraging the IGDT, guarantees that the anticipated demand falls within the range of fluctuations. A decision model inclined towards risk aversion, framed around the IGDT robust function, is articulated as follows:

26 $$\begin{aligned} & \max \,(\alpha_{{{\text{Re}} n}}^{c} ,\alpha_{Ld}^{c} ) \\ & subjected\,to\,\,OF_{Robust} \le (1 + \sigma )OF_{Deterministic} ,\,\sigma \in [0,1) \\ & P_{{{\text{Re}} n,t}} = (1 - \alpha_{{{\text{Re}} n}}^{c} )\overline{P}_{{{\text{Re}} n,t}} \\ & P_{Ld,t} = (1 + \alpha_{Ld}^{c} )\overline{P}_{Ld,t} \\ \end{aligned}$$

where, $\overline{P}_{{{\text{Re}} n,t}}$ and $\overline{P}_{Ld,t}$ respectively are the greatest power size of energy units and demand.

**Fig. 3:**
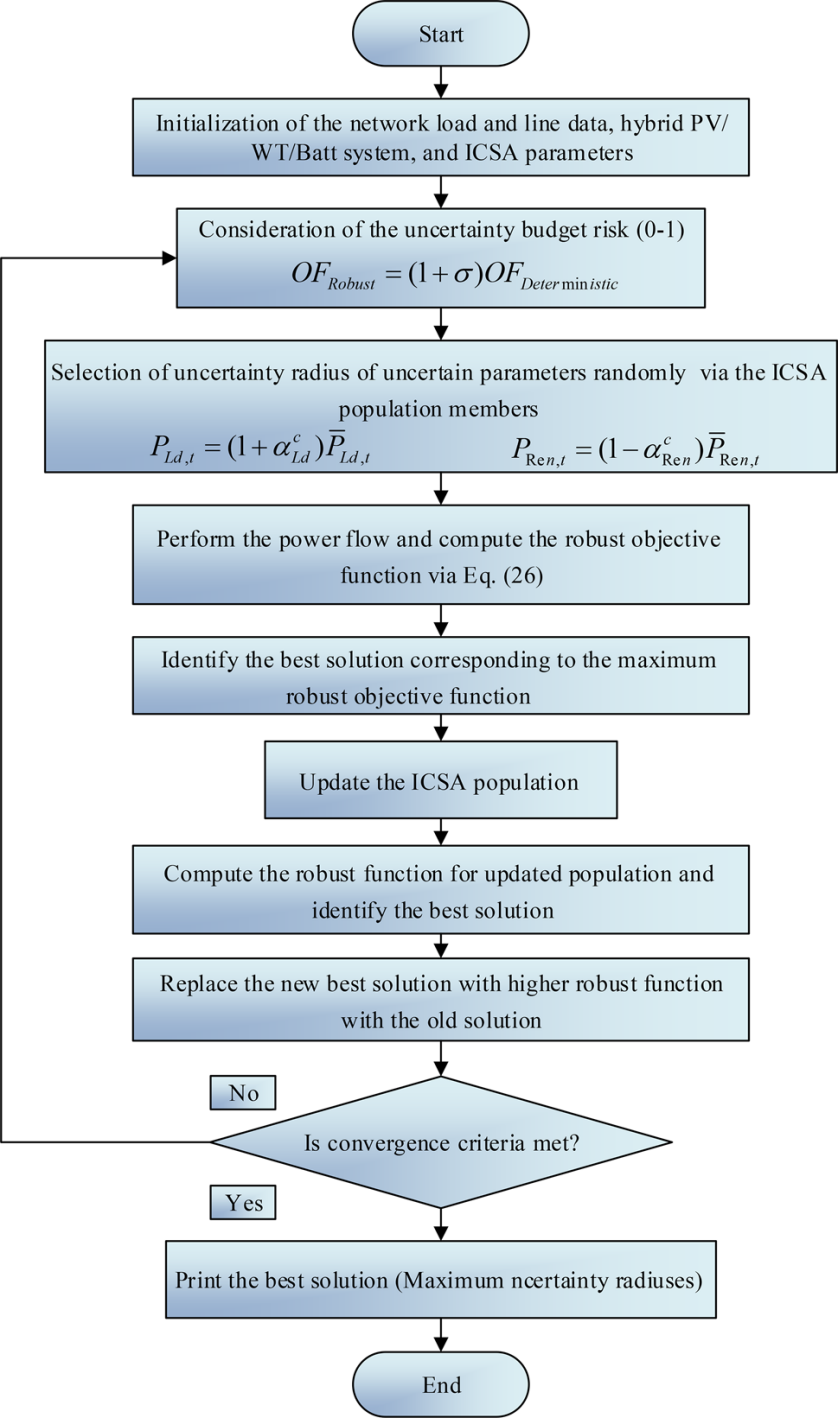
Flowchart of the solving-problem based on the IGDT.

The flowchart of the solving-problem based on the IGDT is depicted in Fig. 3.

## Simulation results and discussion

**Fig. 4:**
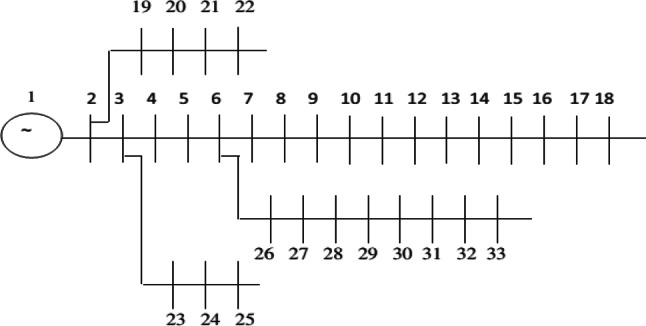
Single-line schematic of 33-bus network.

**Fig. 5:**
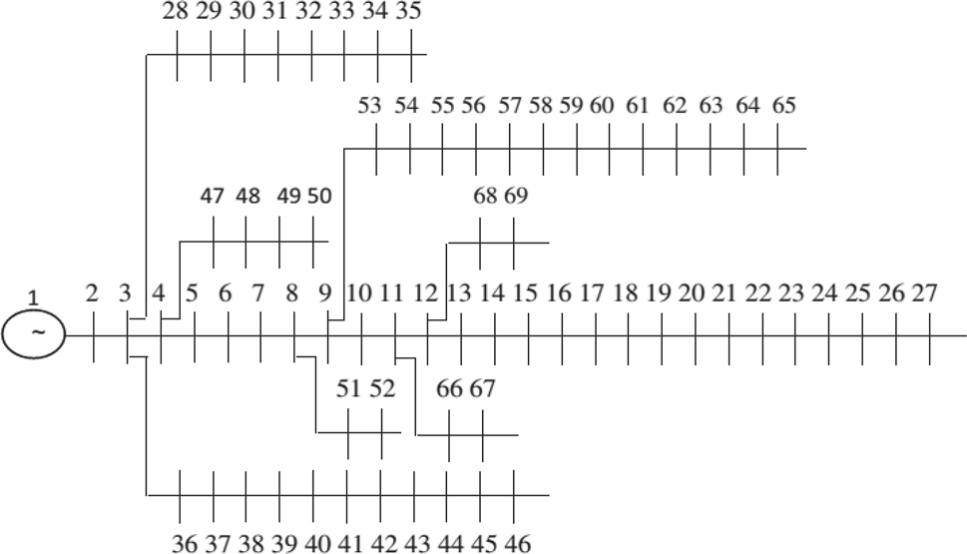
Single-line schematic of 69-bus network.

The outcomes from simulating the allocation of the HS in the networks, targeting a decrease in active losses, voltage condition enhancement, and boosted reliability for consumers facing network line failures, utilizing a multi-objective strategy. This methodology is developed on two 33 and 69 bus networks. Figure 4 displays the single-line schematic of the 33-bus radial network. Information regarding demand and lines for the 33-bus network is derived from^41^, while data on reliability, such as line failure rates and restoration times, come from^42^. The network includes a primary branch alongside three sub-branches, with the total network bearing a load of 3.72 MW and 2.3 MVAr. The alternative network explored is the 69-bus radial network, illustrated in Fig. 5, featuring main feeders plus seven sub-branches. The 69-bus network supports a total demand of 3.802 MW and 2.696 MVAr, with detailed information provided in^43^. The control parameter of CSA such as AP, and FL are considered 0.1, and 2 according to the its reference paper^40^. Also, the decision variables vector includes the install location of the hybrid energy system (buses 2–33 for 33-bus network, and buses 2–69 for 69-bus network), and also size of the PVs (0–1000), WTs (0–1000), and batteries (0–3000) number are are bound.

**Fig. 6:**
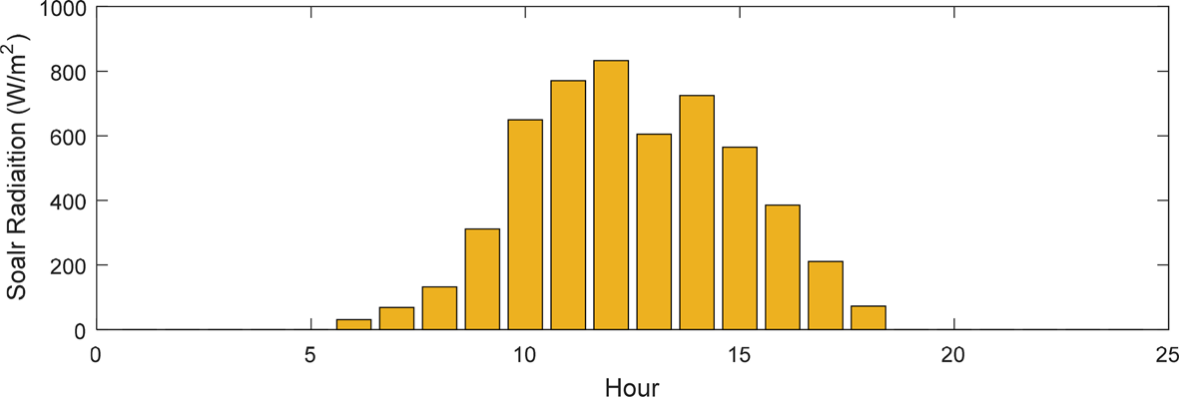
Solar radiation profile for a day.

**Fig. 7:**
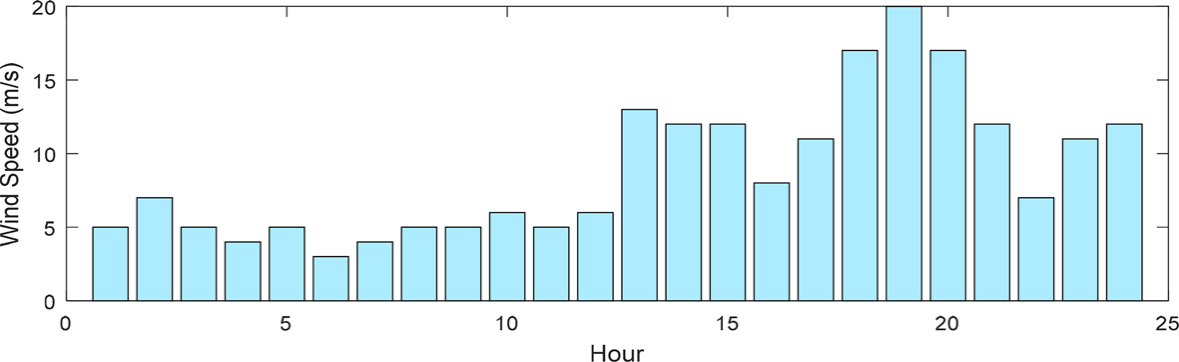
Wind speed profile for a day.

**Fig. 8:**
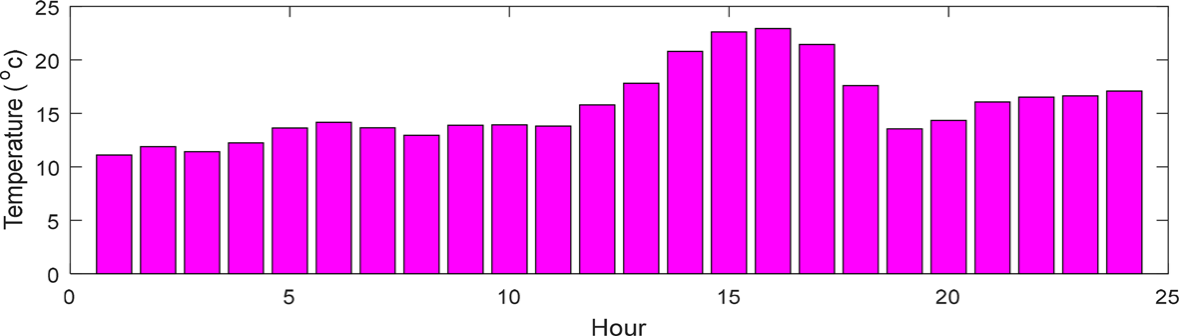
Temperature profile for a day.

**Fig. 9:**
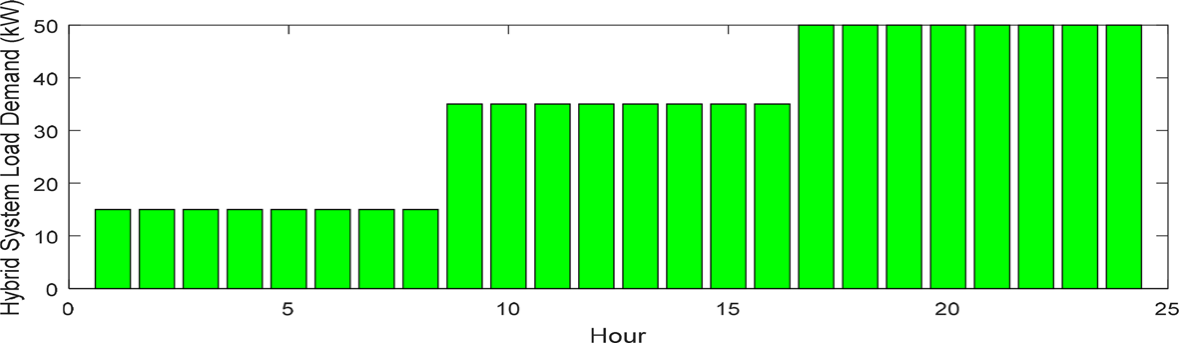
HS load profile for a day.

This research presents data on solar radiation intensity, wind speed, temperature, and the needed power for the HS demand over a 24-h, illustrated in Figs. 6, 7, 8 and 9. The meteorological data pertains to Gorgan city^3,44^. Additionally, Table 1 outlines the techno-economic information of the HS devices.

**Table 1:** Techno-economic information of the HS devices^3,35–37^.

Device	Item	Value
PV	Rated power	1 kW
	Lifetime	20 yrs
	Investment cost	2000$
	Cost of replacement	–
	Cost of O&M	33$/ year
WT	Rated power	1 kW
	vci	3 m/s
	vr	13 m/s
	vco	20 m/s
	Lifetime	20 yrs
	Investment cost	3200$
	Cost of replacement	–
	Cost of O&M	100$/ year
Battery	Maximum size	1 kA h
	Maximum size	0.2 kA h
	Charge efficincy	0.9
	Discharge efficincy	0.9
	DOD	0.8
	Lifetime	5 yrs
	Investment cost	100
	Cost of replacement	–
	Cost of O&M	5$/ year
Inverter	Efficiency	1

### Results of deterministic approach

#### 33-bus network

**Fig. 10:**
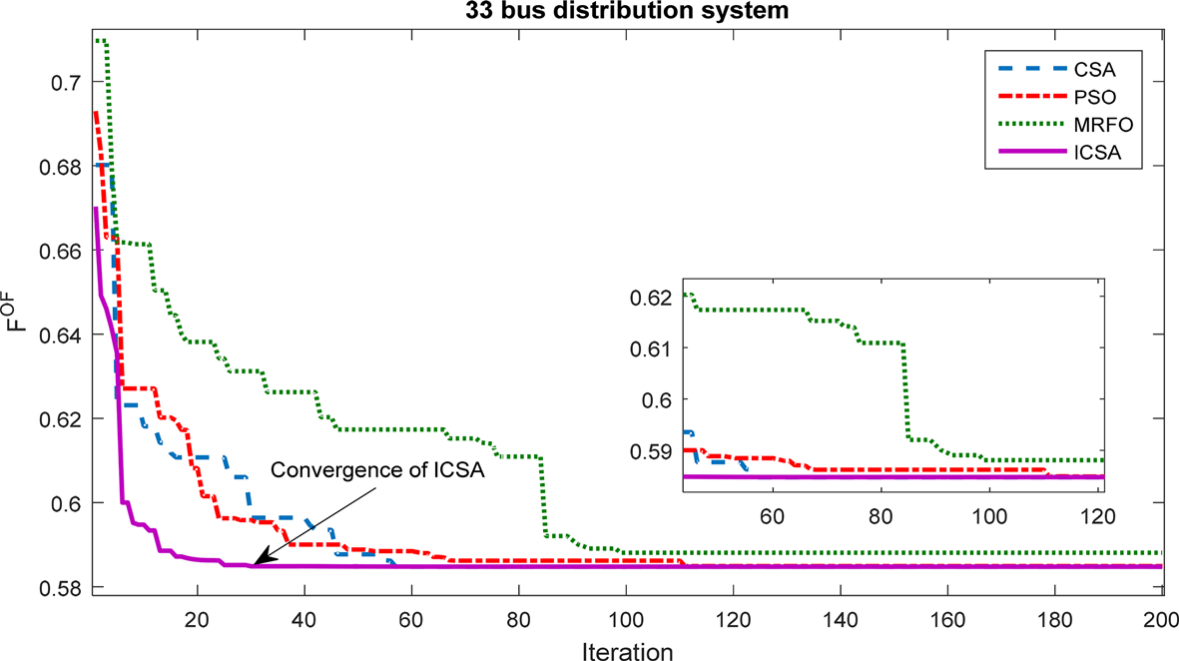
Convergence process of methods in the allocation of HS in the 33-bus network.

This section discusses the outcomes of employing a multi-objective approach to design and place a PV/WT/Batt system within the 33 bus network. The primary objectives include reducing losses, minimizing voltage deviations across network buses, and optimizing the HS cost. Figure 10 illustrates the convergence process of the proposed ICSA in comparison to the CSA, PSO, and MRFO for solving this problem. It is evident that the ICSA exhibits fewer convergence tolerance and achieves convergence more rapidly than the other methods. Following the ICSA, the CSA demonstrates superior performance in optimizing the problem.

Table 2 provides the quantitative outcomes of implementing the HS within the 33-bus network via the ICSA, CSA, PSO, and MRFO. It includes the optimal capacities of PVs, WTs, and battery banks, along with the system’s installation locations in the network. Additionally, the table presents the loss, bus voltage deviations, minimum voltage, cost of loss, financial benefit, and HS costs.

**Table 2:** The results of HS allocation in 33-bus network.

Item	Initial	ICSA	CSA	MRFO	PSO
$$N_{PV}$$	–	802	803	803	803
$$N_{WT}$$	–	673	672	674	672
$$N_{Batt}$$	–	2866	2897	2742	2650
Site	–	Bus 30	Bus 30	Bus 30	Bus 30
Active loss (kW)	202.68	113.88	113.92	114.16	113.98
Voltage deviation (p.u)	0.029862	0.0183	0.0184	0.0185	0.0184
OF	–	0.5846	0.5848	0.5880	0.5850
Voltage minimum (p.u)	0.91308	0.9384	0.9382	0.9378	0.9381
Losses cost ($)	106,530	59,855	59,876	60,002	59,907
Saving ($)	–	46,675	46,654	46,528	46,623
HS Cost (M$)	––	2.43880	2.43887	2.43901	2.43851
PV Cost (M$)		0.41207	0.41212	0.41214	0.41214
WT Cost (M$)	–	1.8917	1.89295	1.8930	1.8924
Batt Cost (M$)	–	0.13503	0.13393	0.13387	0.13397

**Fig. 11:**
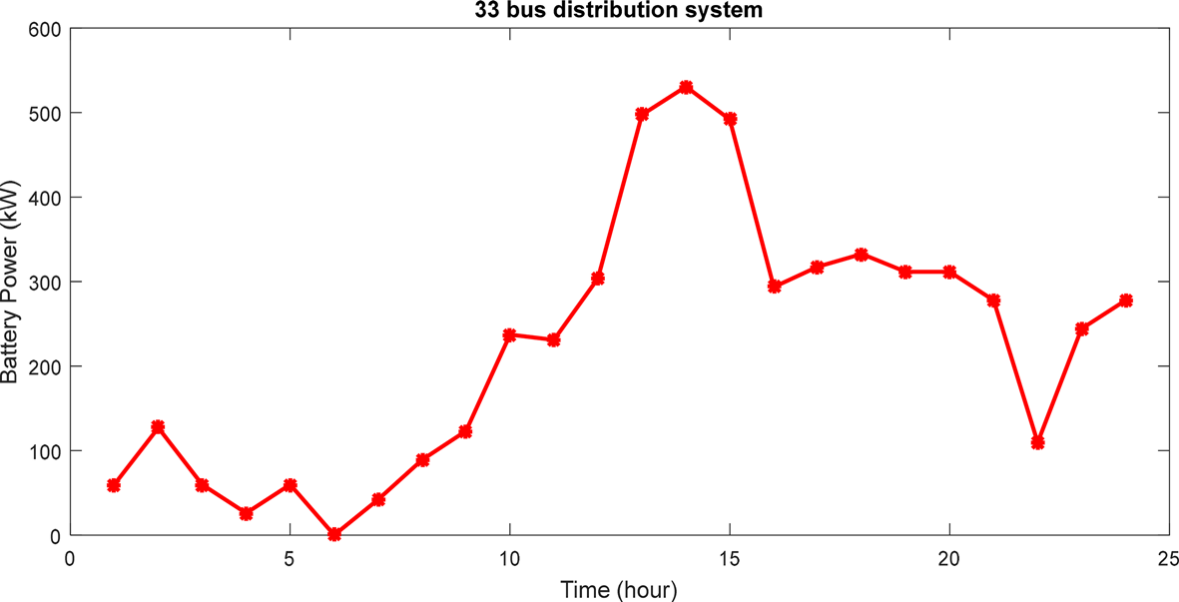
The battery power variations for 33-bus network for 24 h.

Analysis of Table 2 reveals that the ICSA outperforms other techniques in both system design and placement within the network, resulting in lower power losses and voltage deviations. Furthermore, compared to alternative methods, the ICSA approach achieves greater minimum voltage, lower cost of loss, and more financial profit. The table also details the costs associated with supplying the system load, losses reduction, and enhancing the network voltage profile. Notably, while all three methods yield similar overall costs, it’s important to clarify that the costs of the HS are not directly incorporated into the problem target function. Instead, these costs are calculated based on the optimal equipment capacities of the HS. These findings indicate that the proposed method exhibits a faster convergence rate than other methods. Figure 11 illustrates the power curve of the battery bank over a 24-h period using the ICSA. The ascending mode depicts the battery bank’s charging phase, while the descending mode signifies its discharging phase to fulfill the HS load requirements.

**Fig. 12:**
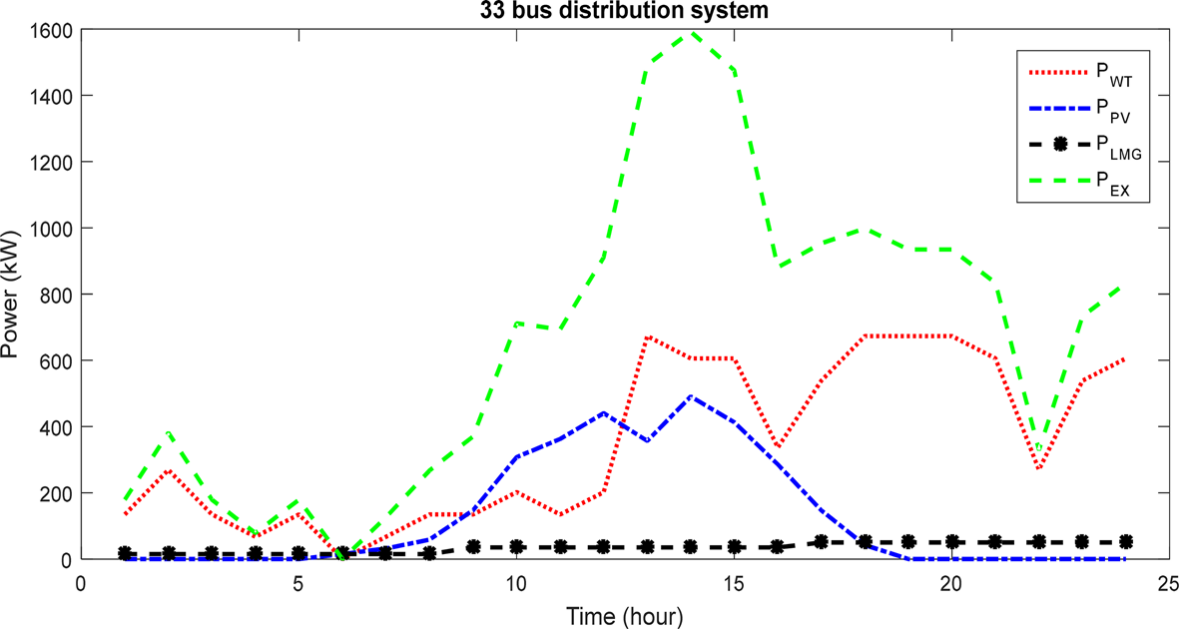
The different components contribution for 33 bus-network for 24 h via ICSA.

Figure 12 depicts the participation curve of units and power management over a 24-h period via the ICSA within the network. The graph illustrates variations in the generation power of PVs and WTs, the demand of the HS, and variations in excess power. The HS’s demand is sulppied through the combination of PVs and WTs, as well as the battery energy management. Additionally, surplus power is transferred to the network to mitigate losses and voltage oscillations across network buses, specifically targeting bus 30.

**Fig. 13:**
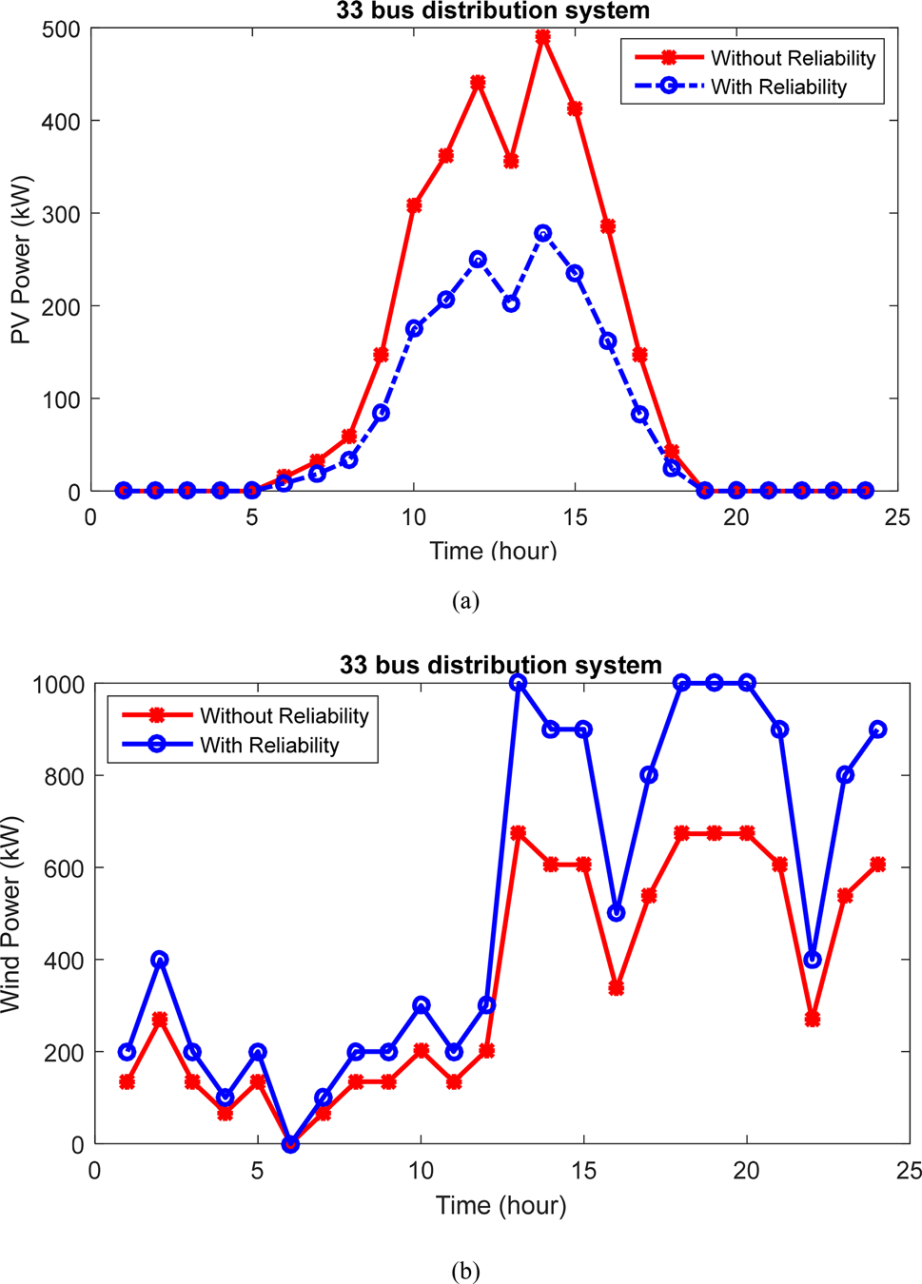
Production power changes of (**a**) PVs and (**b**) WTs with and without considering the reliability in the 33 bus network.

Table 3 presents the outcomes of designing and planning a HS in the 33-bus network. The aim is to minimize the losses, voltage oscillations, and enhance network reliability amidst line withdrawals and unavailability. The results compare scenarios with and without considering reliability goals, assessing their impact on each objective. Initially, the ENS reliability index stands at 6.44 MWh. Without prioritizing reliability, this index improves to 4.317 MWh with HS planning. However, by incorporating reliability into the objective function, the ENS reduces further to 1.825 MWh, marking a 71.69% improvement over the baseline and 57.73% compared to the non-reliability scenario. Thus, with reliability as an objective, the program focuses more on meeting subscriber load demands. Nevertheless, this shift also leads to increased power losses from 113.88 kW to 129.17 kW and decreased voltage deviations from 0.0183 to 0.0170. Figure 13 illustrates changes in PV and WT power production over 24 h, highlighting the significant contribution of wind power in enhancing network characteristics.

**Table 3:** The results of considering reliability for 33-bus network.

Item	Without reliability	With reliability
$$N_{PV}$$	802	456
$$N_{WT}$$	673	1000
$$N_{Batt}$$	2866	2759
Site	Bus 30	Bus 30
Active loss (kW)	113.88	129.17
Voltage deviation (p.u)	0.0183	0.0170
ENS (MWh)	4.317	1.825
OF	0.5846	0.5833
Voltage minimum (p.u)	0.9384	0.9434
Losses cost ($)	59,855	67,891
Saving ($)	46,675	38,639
HS Cost (M$)	2.43880	3.1794
PV Cost (M$)	0.41207	0.23420
WT Cost (M$)	1.8917	2.8125
Batt Cost (M$)	0.13503	0.13267

#### 69-busnetwork

**Fig. 14:**
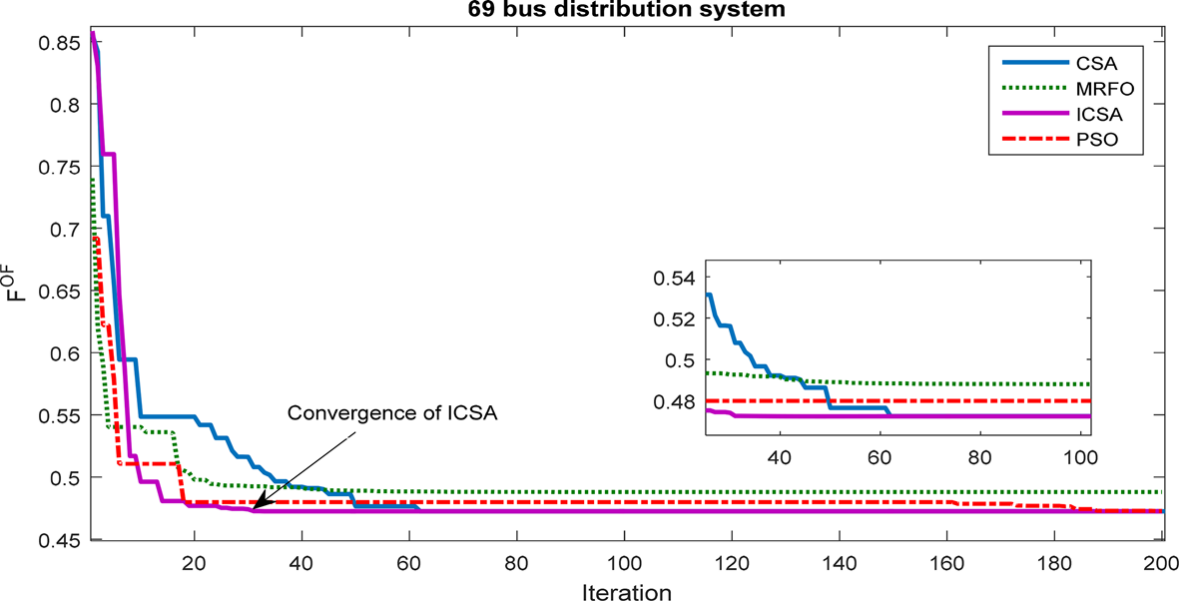
Convergence process of methods for 69-bus network.

In this segment, we showcase the outcomes of addressing the allocation problem of the HS within a different test system, specifically the 69-bus radial network. Figure 14 illustrates the ICSA convergence process the CSA, PSO, and MRFO. It’s evident that the ICSA method demonstrates fewer convergence fluctuations compared to other approaches and achieves convergence more swiftly.

The outcomes of deploying the HS within the 69-bus network through the utilization of ICSA, CSA, PSO, and MRFO are outlined in Table 4.

**Table 4:** The results of HS allocation in 69-bus network.

Parameter	Base	ICSA	CSA	MRFO	PSO
$$N_{PV}$$	–	800	801	801	801
$$N_{WT}$$	–	703	705	706	702
$$N_{Batt}$$	–	2865	2973	2981	2865
Site	–	Bus 61	Bus 61	Bus 61	Bus 61
Active loss (kW)	224.98	102.98	103.18	103.42	103.11
Voltage deviation (p.u)	0.02703	0.0132	0.0133	0.0133	0.0133
OF	–	0.4722	0.4728	0.4872	0.4768
Voltage minimum (p.u)	0.9091	0.9649	0.9646	0.9645	0.9647
Losses cost ($)	118,249	54,126	54,231	54,357	54,194
Saving ($)	–	64,123	64,018	63,892	64,055
HS Cost (M$)	–	2.8301	2.8279	2.8336	2.8276
PV Cost (M$)	–	0.4100	0.4124	0.3972	0.4110
WT Cost (M$)	–	2.285	2.2775	2.299	2.279
Batt Cost (M$)	–	0.1351	0.1380	0.1374	0.1376

**Fig. 15:**
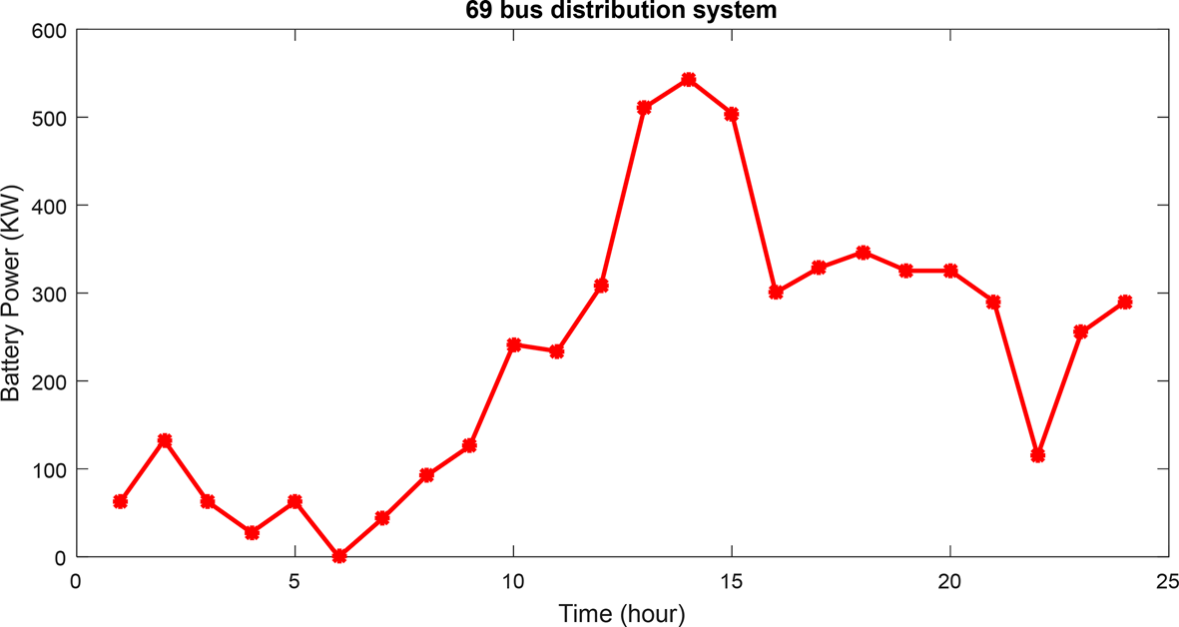
The battery energy variations for 69-bus network for 24 h.

As given in Table 4, the ICSA determines bus 61 for the installation of the HS, contrasting with the traditional CSA, PSO, and MRFO. Notably, the ICSA approach for sizing and siting the HS within the grid results in reduced power loss and voltage deviation. Comparatively, it achieves a lower minimum voltage, decreased cost of losses, and higher financial savings compared to alternative methods. Specifically, using the ICSA yields a loss of 102.98 kW, a voltage deviation of 0.0132 perunits, a minimum voltage value of 0.9649 perunits, and financial savings amounting to $64,123. The design costs for the HS via the ICSA, CSA, PSO, and MRFO are calculated as M$2.8301, M$2.8279, and M$2.8336, and M$2.8276 respectively. Figure 15 illustrates the battery bank’s power variations over 24 h using the ICSA method, showcasing the fluctuations in charge and discharge cycles.

**Fig. 16:**
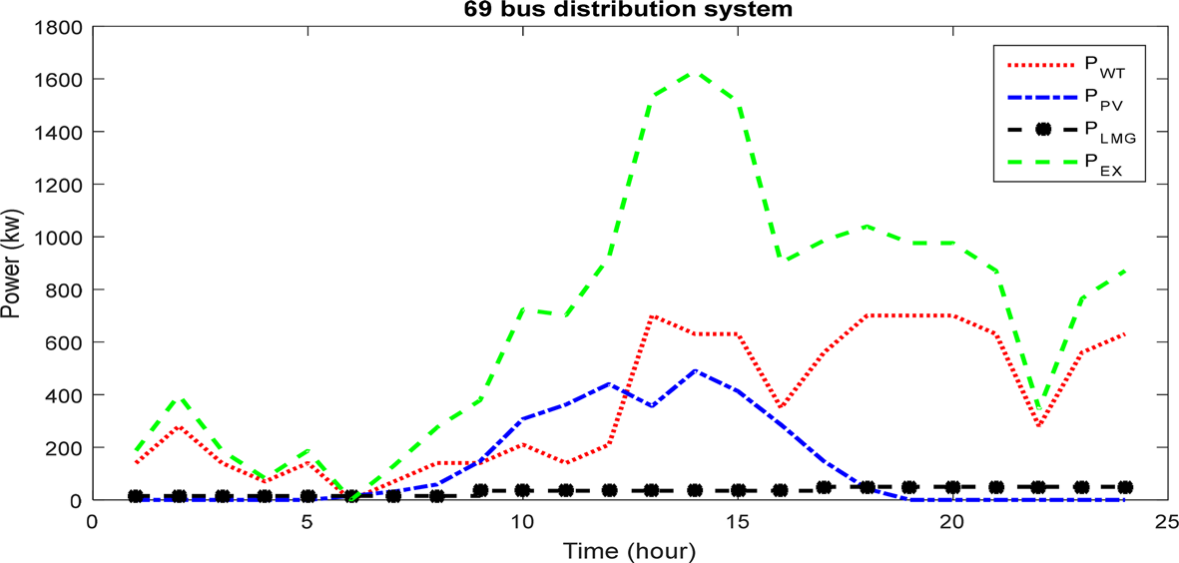
The different components contribution for 69-bus network for 24 h via ICSA.

Figure 16 displays the production power of renewable resources, excess power, and the HS power over a during a fully day, utilizing the ICSA within the 69 bus network.

**Fig. 17:**
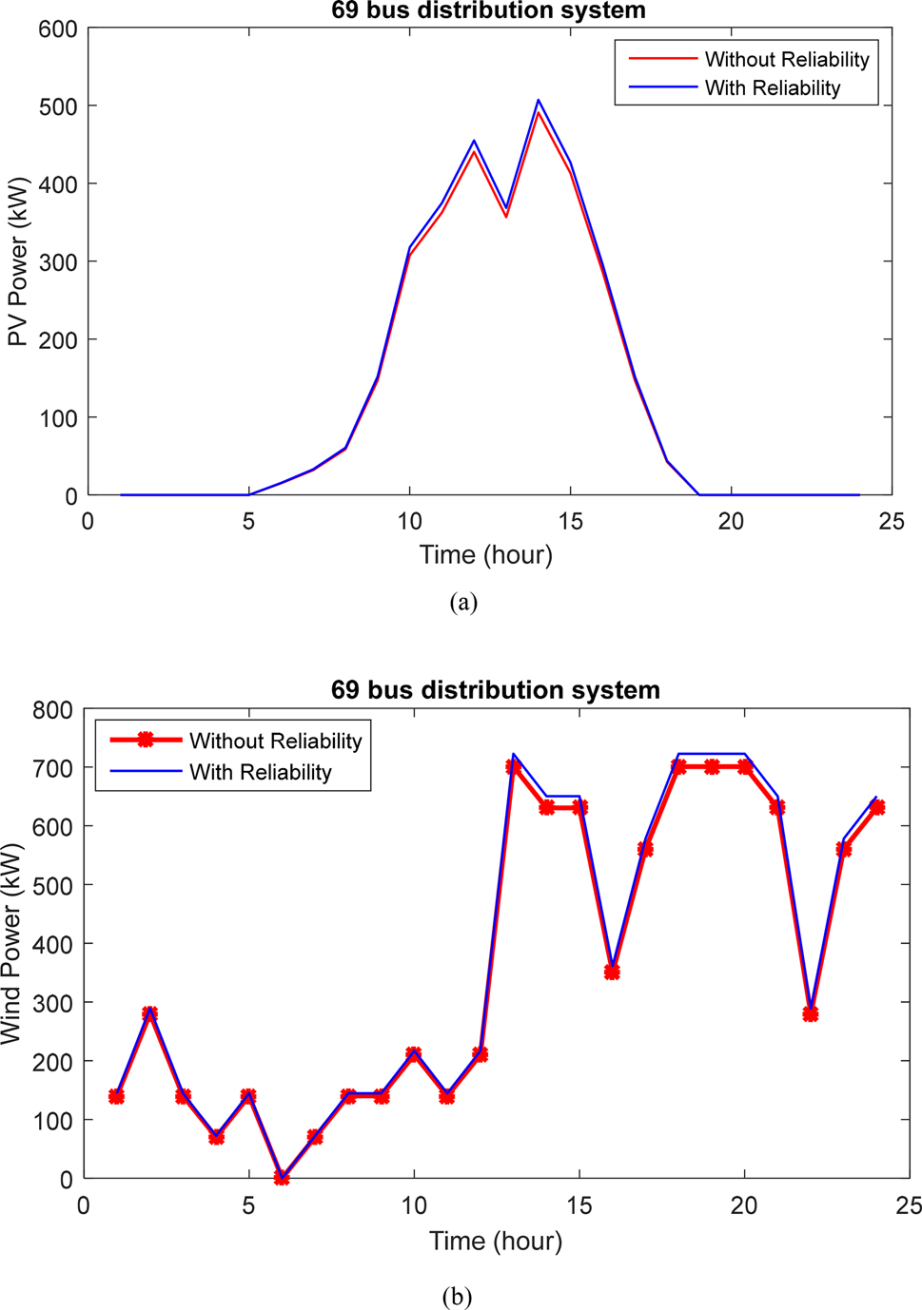
Production power changes of (**a**) PVs and (**b**) WTs with and without considering the reliability in the 69 bus network.

The results of designing and planning the HS in the 69-bus network. The primary aim is to minimize the losses and voltage oscillations while enhancing network reliability amidst line outages and unavailability. Table 5 provides a comparison of results with and without considering reliability goals to assess its impact on each objective. The optimization program yields 821 panels, 723 turbines, and 914 batteries. Initially, the reliability index (ENS) stands at 13.94 MWh. Without prioritizing reliability, this index improves to 10.17 MWh with HS planning. However, by incorporating reliability into the objective function, the ENS reduces further to 3.82 MWh, marking a 72.60% improvement over the baseline and 32.94% compared to the non-reliability scenario. Thus, with reliability as an objective, subscriber load provisioning is more reliable. However, this shift also increases power loss from 103.09 kW to 113.76 kW, and voltage deviations are without any change. Figure 17 illustrates changes in PV and WT power production over 24 h, highlighting the significant contribution of wind power in improving network characteristics.

**Table 5:** The results of considering reliability for 69-bus network.

Item	Without reliability	With reliability
$$N_{PV}$$	800	821
$$N_{WT}$$	703	723
$$N_{Batt}$$	2865	914
Site	Bus 61	Bus 62
Active loss (kW)	103.09	113.76
Voltage deviation (p.u)	0.0132	0.0132
ENS (MWh)	10.17	3.82
OF	0.4722	0.4703
Voltage minimum (p.u)	0.9649	0.9664
Losses cost ($)	54,126	59,792
Saving ($)	64,123	58,457
HS Cost (M$)	2.8301	2.81210
PV Cost (M$)	0.4100	0.42160
WT Cost (M$)	2.285	2.34870
Batt Cost (M$)	0.1351	0.04180

### Results of robust approach with uncertainty risk

In this segment, we present the outcomes of robust optimization for HS across both 33- and 69-bus networks. This optimization accounts for the risk of uncertainty by employing information gap decision theory in conjunction with the ICSA solver. For each network, the system’s resilience against prediction errors in the worst-case scenario of uncertainty has been evaluated.

#### 33-bus network results

**Fig. 18:**
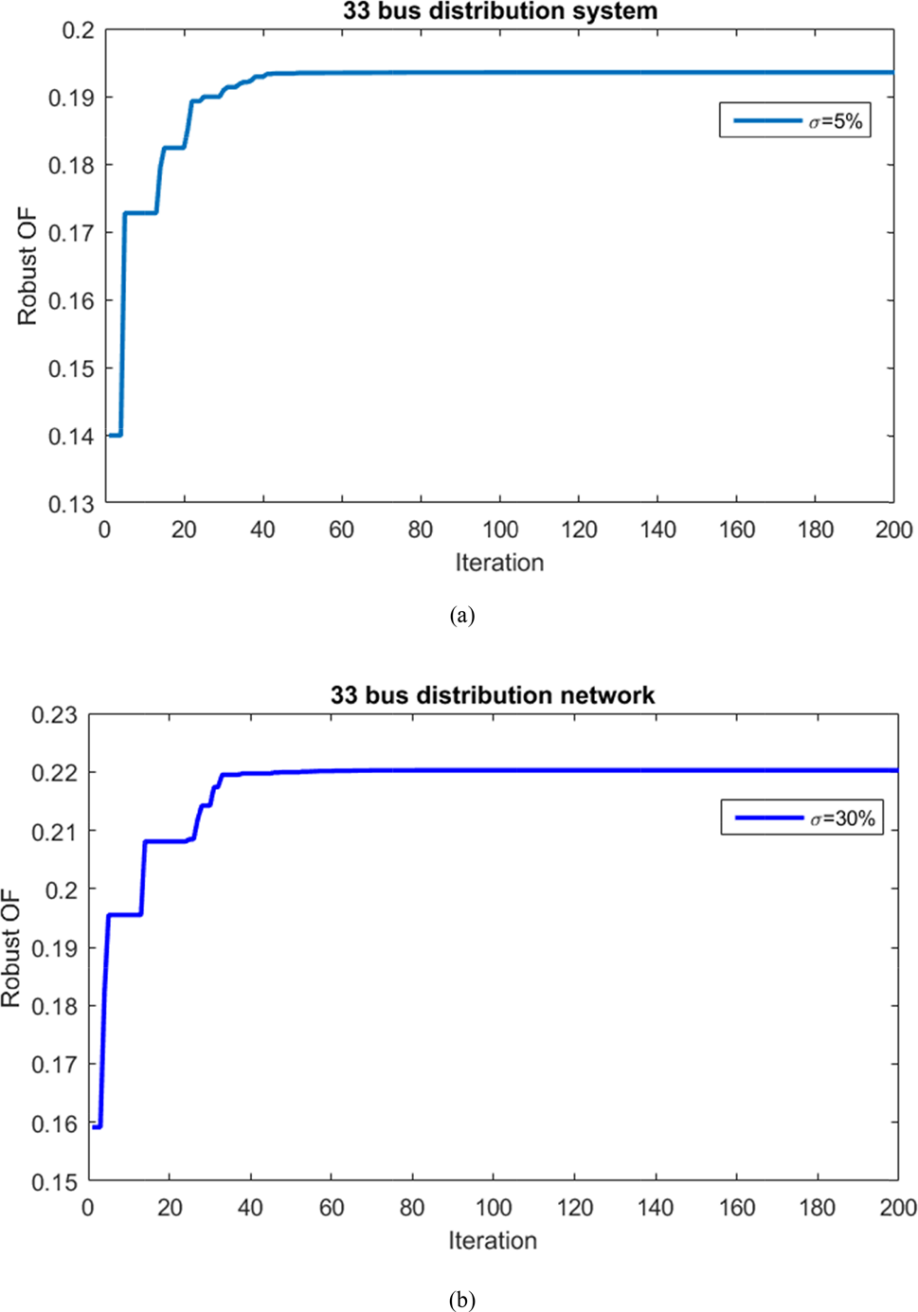
Convergence curves of robust optimization problem of HS for 5% and 30% uncertainty budgets for 33-bus network.

The outcomes of the robust optimization for the HS is given for a 33-bus network. The goal is to maximize the MUR of renewable production and network demand, employing the information gap decision theory approach alongside the ICSA solver. Robust optimization is conducted across various uncertainty budgets, revealing that problem resilience to uncertainties diminishes for budgets below 5% and above 30%. Consequently, the convergence curves for robust optimization of the HS are provided for uncertainty budgets of 5% and 30%, as depicted in Fig. 18. The robust objective function corresponds to the multi-objective function of simultaneously maximizing the MURs. The convergence process highlight the optimal convergence speed of the IFDA in solving the problem and attaining the best solution.

Table 6 presents the MUR for resource production and demand in the robust optimization of the hybrid PV/WT/Batt system within the 33-bus network. For uncertainty budgets of 5% and 30%, the uncertainty radius values for renewable resource production are 19.79% and 44.53%, respectively. Likewise, the uncertainty radius values for network load demand are 19.30% and 22.18%, respectively. Essentially, these maximum uncertainty radius values determine the system’s resistance level under conditions of uncertainty risk, while ensuring adherence to network operation constraints.

**Table 6:** Values of the MUR of resource production and load considering σ = 3% and σ = 30% for 33-bus network.

Uncertainty Radius/Uncertainty Budget	σ = 5% (%)	σ = 30% (%)
Renewable Generation	19.79	44.53
Network Load	19.30	22.18

The numerical results for each objective in both deterministic and robust optimization problems, considering uncertainty budgets of 5% and 30% for the 33-bus network, are shown in Table 7. As uncertainty increases, compared to the target function in the deterministic case, the adverse effects of reduced resource production and increased load demand cause the values of each objective in the robust optimization to decrease compared to the deterministic approach without uncertainty. When the uncertainty budget rises from 5 to 30%, active power losses increase from 134.75 kW to 145.35 kW, voltage deviations rise from 0.0192 p.u. to 0.0237 p.u., and unsupplied energy grows from 1.867 MWh to 1.920 MWh. Meanwhile, the minimum network voltage drops from 0.9388 p.u. to 0.9348 p.u.

**Table 7:** Numerical results of different goals in the deterministic and robust problem considering σ = 5% and σ = 30% for 33-bus network.

Item/Approach	Deterministic	MCS^3^	Robust (σ = 5%)	Robust (σ = 30%)
_Active loss (kW)_	129.17	70–120	134.75	145.35
_Voltage deviation (p.u)_	0.017	0.01–0.0275	0.0192	0.0237
_ENS (MWh)_	1.825	0.25–6	1.867	1.920
_Voltage minimum (p.u)_	0.9434	–	0.9388	0.9348

**Fig. 19:**
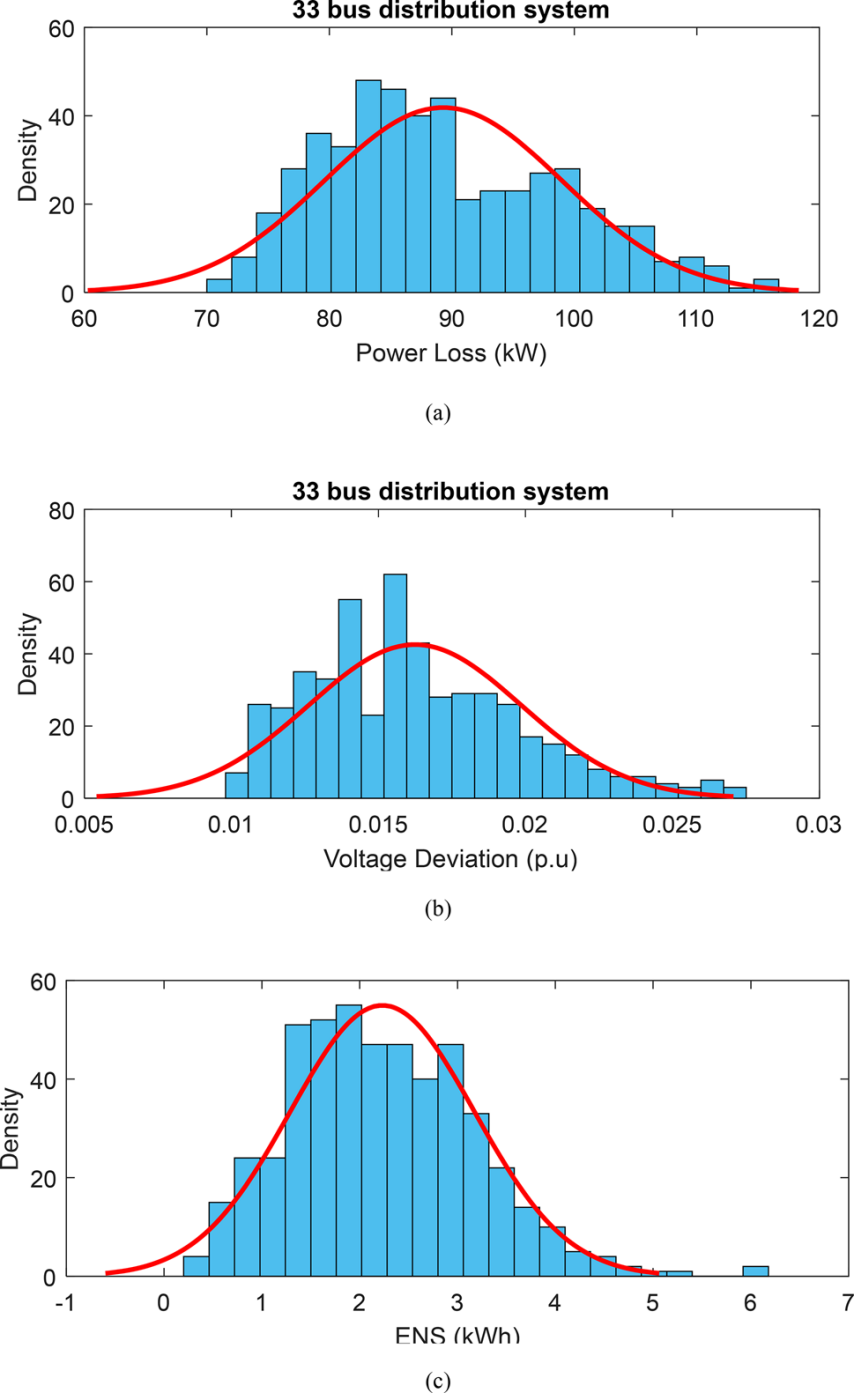
PDF (**a**) active losses (**b**) voltage deviations and (**c**) ENS after HS allocation^3^.

Additionally, robust optimization outcomes are compared with probabilistic methods using Monte Carlo simulations. Wind speed, solar radiation, and network load demand uncertainties are modeled using Weibull, beta, and normal probability distributions (PDFs), respectively. For network load, a normal distribution with an average of 85% and a standard deviation of 25% is assumed for each bus. The PDFs for active power losses, voltage deviations, and the network’s ENS, derived from applying the Harmony Search (HS) algorithm to the 33-bus network via ICSA, are shown in Fig. 19. For uncertainty in power losses, voltage deviations, and the ENS index, the total weighted values (each scenario’s value multiplied by its probability) are calculated. Specifically, active losses are 106.70 kW, voltage deviations are 0.0165 p.u., and ENS is 1.634 MWh. An analysis of potential outcomes and the corresponding probability distributions in Fig. 19 shows active losses between 70 and 120 kW, voltage deviations between 0.01 p.u. and 0.0275 p.u., and unsupplied energy ranging from 0.25 MWh to 6 MWh, each occurring with a specific probability. It becomes evident that the Monte Carlo random method is insufficient in providing robust solutions for all objectives under uncertainty, while the robust approach consistently achieves the MUR for both resources and demand across different uncertainty budgets.

**Fig. 20:**
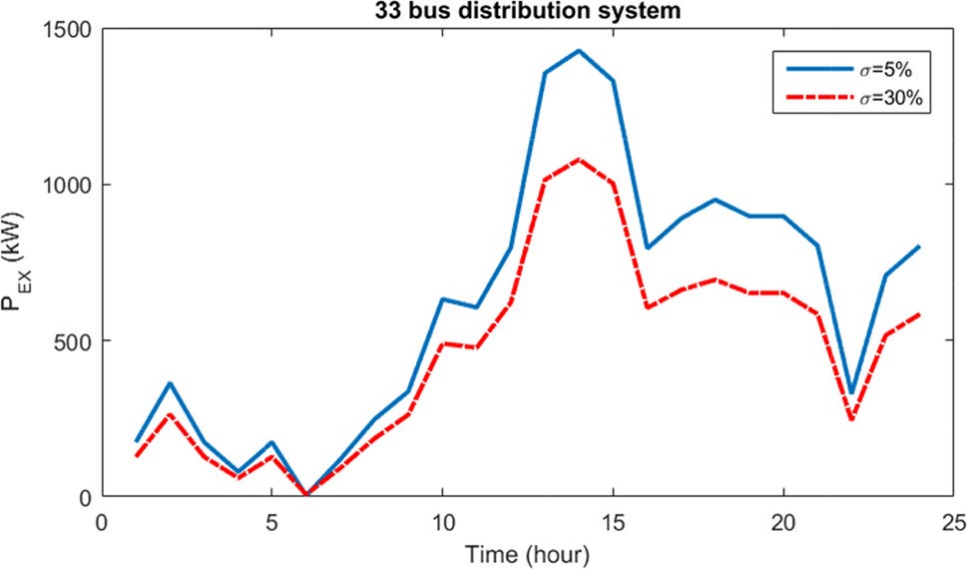
Curve of power changes transferred to the network by HS for 5% and 30% uncertainty budgets for 33-bus network.

Figure 20 displays the curve illustrating the fluctuations in power transferred to the grid by the HS for uncertainty budgets of 5% and 30%. It’s evident that as the risk associated with the uncertainty budget increases, there’s a noticeable reduction in the amount of power injected to the grid by the HS. This decrease correlates with further declines in resource production and increases in load demand.

#### 69-bus network results

**Fig. 21:**
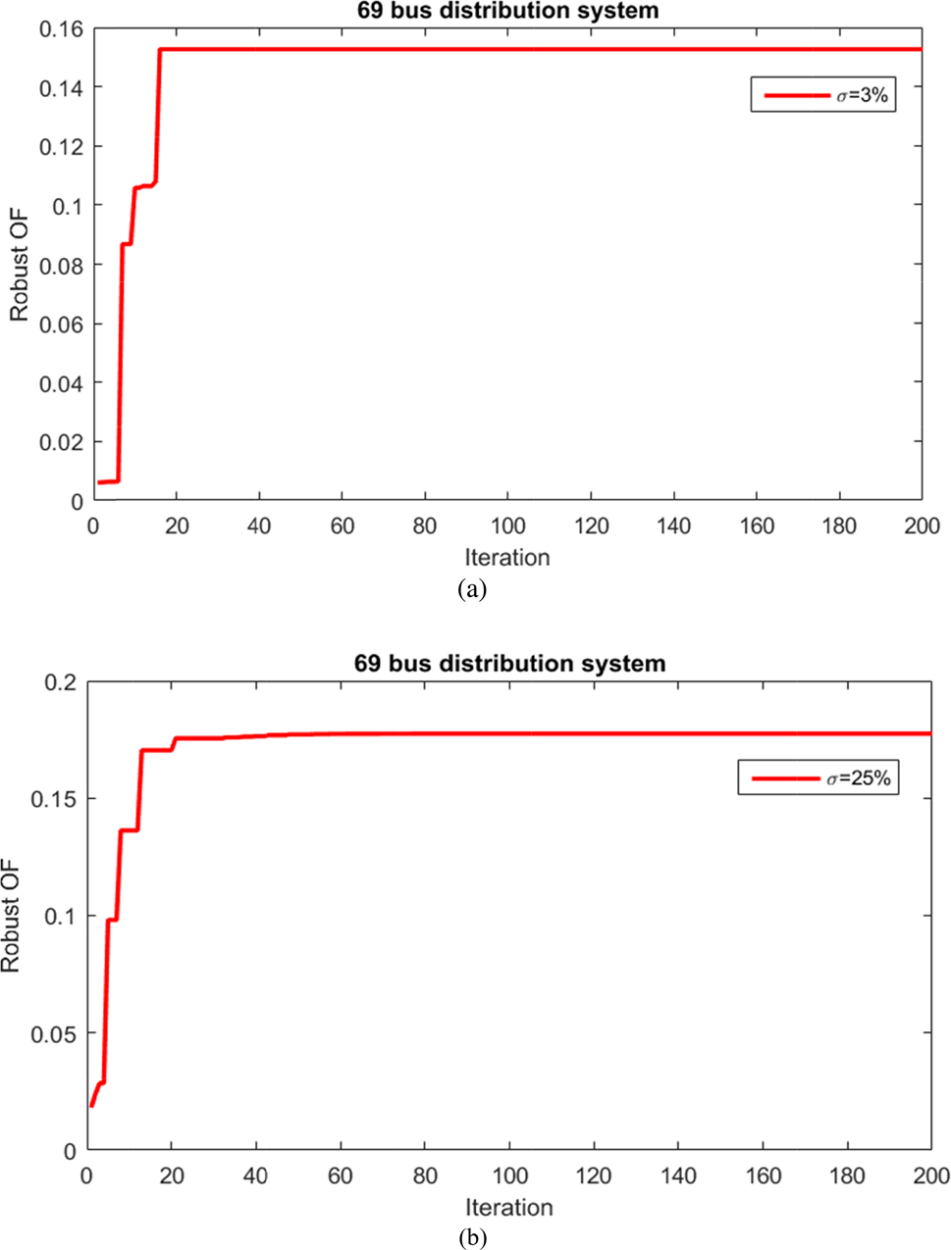
Convergence curves of robust optimization problem of HS for 3% and 25% uncertainty budgets for 69-bus network.

The outcomes of a robust process utilizing the information gap decision theory approach and the ICSA solver for optimizing the HS within the 69-bus network are given to maximize the MUR associated with renewable resource producton and network demand. The robust optimization procedure has been thoroughly detailed, and it has been implemented across various uncertainty budgets. The results indicate that the problem tends to violate operational constraints for uncertainty budget values below 3% and above 25%. Figure 21 illustrates the convergence curves of the robust optimization problem for the HS, showcasing results for uncertainty budgets of 3% and 25%. These curves highlight the optimal convergence rate of the ICSA in addressing the problem and attaining the best solution.

Table 8 outlines the numerical outcomes depicting the MUR values concerning source generation and grid load demand in the resilient optimization of the HS within a 69-bus network, considering uncertainty budgets of 3% and 25%. For the uncertainty budget of 3%, the MUR of renewable resources production stands at 27.95%, while for the 25% uncertainty budget, it reaches 36.22%. Similarly, the MUR for the network demand is 13.95% for the 3% uncertainty budget and 16.97% for the 25% uncertainty budget. This indicates the system’s level of resilience under conditions of uncertainty risk while adhering to network operation constraints.

**Table 8:** MUR of resource production and demand considering σ = 3% and σ = 25% for 69-bus network.

Uncertainty Radius/Uncertainty Budget	σ = 3% (%)	σ = 25% (%)
Renewable Generation	27.95	36.22
Network Load	13.85	16.97

Table 9 presents the numerical results for various objectives within both deterministic and robust problem frameworks, considering uncertainty budgets of 3% and 25% for the 69-bus network. As the uncertainty budget increases from 3 to 25%, there is a noticeable rise in power loss from 129.52 kW to 142.92 kW, voltage deviations from 0.0174 per unit to 0.0196 per unit, and unsupplied energy from 7.25 MWh to 8.12 MWh. Additionally, the minimum grid voltage drops from 0.9593 per units to 0.9526 per units.

**Table 9:** Numerical results of different objectives in the deterministic and robust problem considering σ = 3% and σ = 30% for 69-bus network.

Item/Approach	Deterministic	MCS^3^	Robust (σ = 3%)	Robust (σ = 25%)
_Active loss (kW)_	113.76	40–220	129.52	142.92
_Voltage deviation (p.u)_	0.0132	0.0065–0.025	0.0174	0.0196
_ENS (MWh)_	6.82	1–10	7.25	8.12
_Voltage minimum (p.u)_	0.9664	–	0.9593	0.9526

**Fig. 22:**
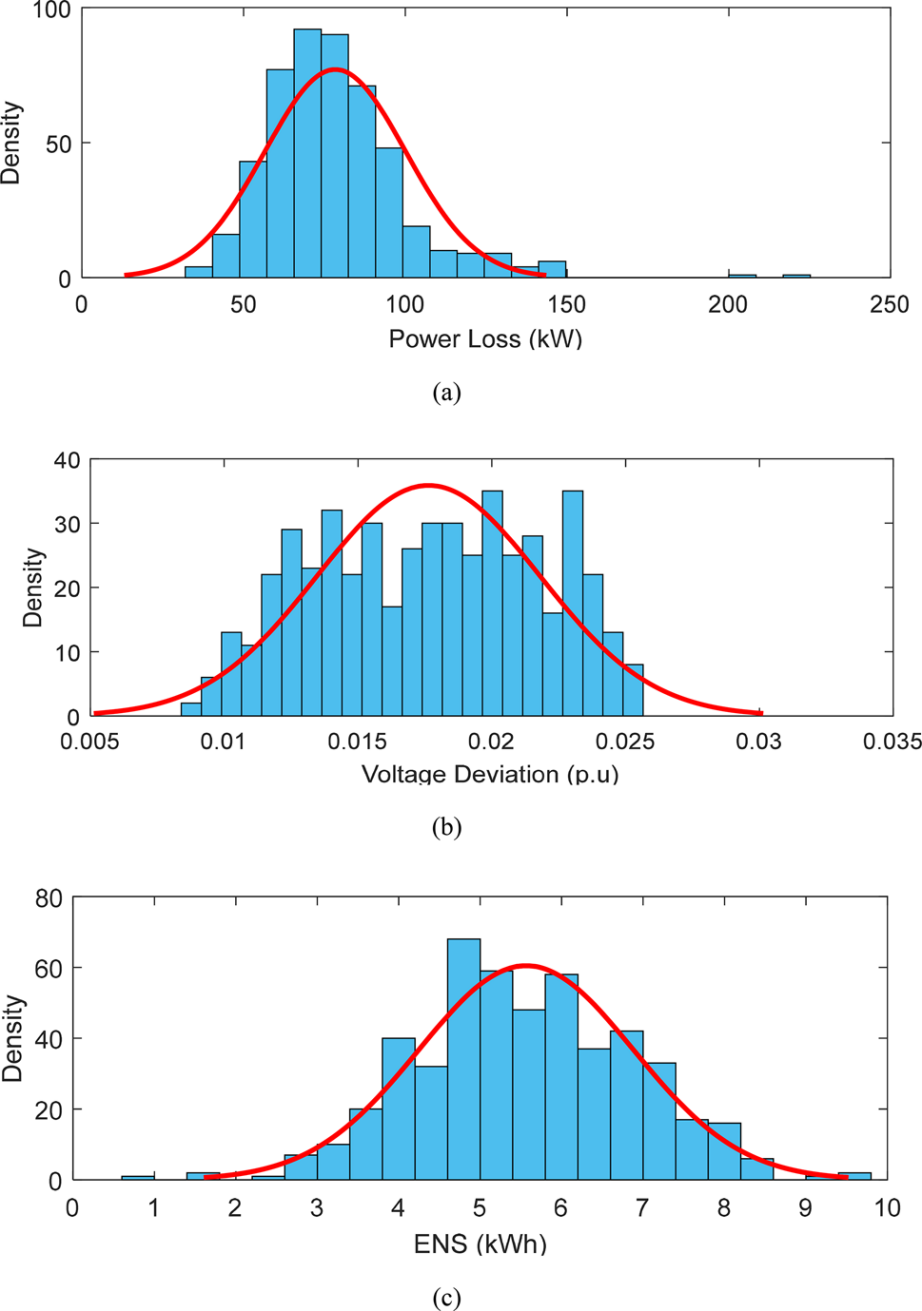
Probability distribution function (**a**) active power losses (**b**) voltage deviations and (**c**) ENS index of 69-bus network after installing the HS^3^.

Furthermore, a comparison is made between the results of stochastic methods using Monte Carlo simulation and robust optimization under uncertainty. Figure 22 shows the probability distribution functions for active power losses, voltage deviations, and network reliability following the optimization of the Harmony Search (HS) algorithm. An analysis of the 69-bus network and the corresponding probability distributions for each objective reveals power losses ranging from 40 to 220 kW, voltage deviations from 0.0065 per unit to 0.025 per unit, and unsupplied energy ranging from 1 to 10 MWh across various scenarios. It is clear that the random Monte Carlo method is inadequate in providing robust solutions for any objectives under uncertainty, as it depends on probability distribution functions. On the other hand, the robust optimization approach effectively determines the MUR for uncertain load demand and renewable resource parameters, even under the most adverse uncertainty scenarios.

**Fig. 23:**
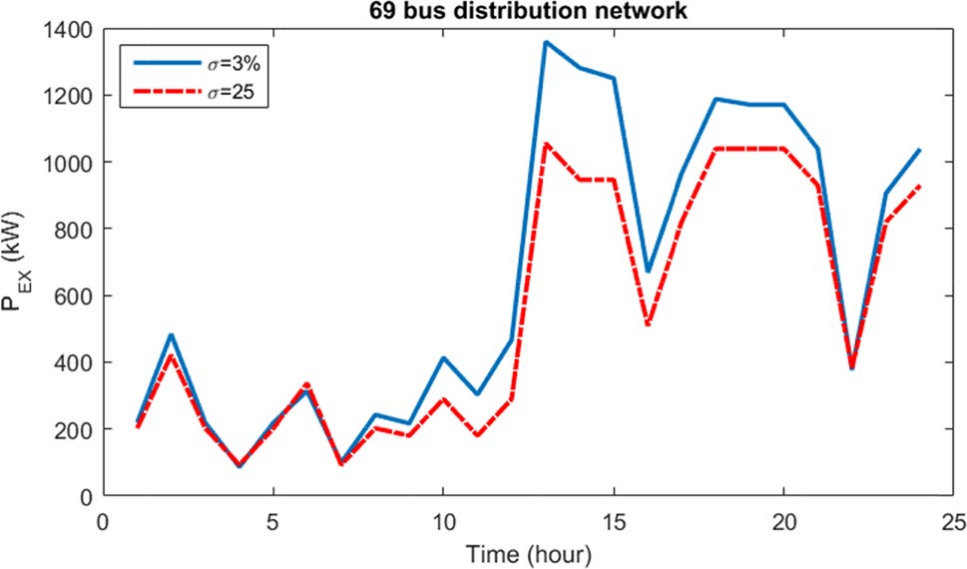
The curve of power changes injected into the grid by the HS for 3% and 25% uncertainty budgets for the 69-bus network.

Figure 23 illustrates the fluctuations in power injected into the grid by the system for uncertainty budgets of 3% and 25%. It is obvious that as the uncertainty budget increases, the amount of power transferred into the network by the HS decreases. This decrease is attributed to the reduction in resource production and the increase in load demand.

## Conclusion

This study presents robust optimization of a hybrid PV/WT/Batt system, aiming to minimize power losses, voltage deviations, and enhance network reliability. Decision variables, including equipment installation location and optimal capacity, were determined using the ICSA and performed on 33 and 69 bus networks.

Key findings include:

- System optimization led to reduced power losses, minimized voltage deviations, and improved network reliability in the networks.
- Resilient optimization of the HS aimed to determine maximum uncertainty radius values for resource generation and network load demand, considering uncertainty risk through the information gap decision theory.
- For the 33-bus network, uncertainty budget values of 5% and 30% resulted in uncertainty radius values of renewable resource production at 19.79% and 44.53%, respectively, and for network load demand at 19.30% and 22.18%, respectively. For the 69-bus network, values were 27.95% and 36.22% for resource production and 13.85% and 16.97% for network load demand, respectively.
- The system’s resistance level under uncertainty conditions was determined based on maximum uncertainty radius values, ensuring compliance with network operation constraints.
- Comparison between random and robust methods revealed that the random Monte Carlo method failed to provide robust solutions under uncertainty, while the robust method effectively attained maximum uncertainty radius values for renewable resources and load demand across various uncertainty budgets.
- Future work is suggested to explore the use of multiple battery-hydrogen storage systems in resilient energy management of microgrids.

## Data Availability

The datasets used and/or analysed during the current study available from the corresponding author on reasonable request.
